# Transcriptional and Microscopic Analyses of Citrus Stem and Root Responses to *Candidatus* Liberibacter asiaticus Infection

**DOI:** 10.1371/journal.pone.0073742

**Published:** 2013-09-13

**Authors:** Valente Aritua, Diann Achor, Frederick G. Gmitter, Gene Albrigo, Nian Wang

**Affiliations:** 1 Citrus Research and Education Center, Department of Microbiology and Cell Science, University of Florida, Lake Alfred, Florida, United States of America; 2 Citrus Research and Education Center, University of Florida, Lake Alfred, Florida, United States of America; 3 Citrus Research and Education Center, Department of Horticultural Sciences, University of Florida, Lake Alfred, Florida, United States of America; University of Torino, Italy

## Abstract

Huanglongbing (HLB) is the most destructive disease that affects citrus worldwide. The disease has been associated with *Candidatus* Liberibacter. HLB diseased citrus plants develop a multitude of symptoms including zinc and copper deficiencies, blotchy mottle, corky veins, stunting, and twig dieback. *Ca*. L. asiaticus infection also seriously affects the roots. Previous study focused on gene expression of leaves and fruit to *Ca*. L. asiaticus infection. In this study, we compared the gene expression levels of stems and roots of healthy plants with those in *Ca*. L. asiaticus infected plants using microarrays. Affymetrix microarray analysis showed a total of 988 genes were significantly altered in expression, of which 885 were in the stems, and 111 in the roots. Of these, 551 and 56 were up-regulated, while 334 and 55 were down-regulated in the stem and root samples of HLB diseased trees compared to healthy plants, respectively. Dramatic differences in the transcriptional responses were observed between citrus stems and roots to *Ca*. L. asiaticus infection, with only 8 genes affected in both the roots and stems. The affected genes are involved in diverse cellular functions, including carbohydrate metabolism, cell wall biogenesis, biotic and abiotic stress responses, signaling and transcriptional factors, transportation, cell organization, protein modification and degradation, development, hormone signaling, metal handling, and redox. Microscopy analysis showed the depletion of starch in the roots of the infected plants but not in healthy plants. Collapse and thickening of cell walls were observed in HLB affected roots, but not as severe as in the stems. This study provides insight into the host response of the stems and roots to *Ca*. L. asiaticus infection.

## Introduction

Huanglongbing (HLB), which is also known as citrus greening, is currently the most destructive disease that affects citrus plants. The disease has been associated with three species of a phloem-limited α-proteobacterium that is designated as *Candidatus* Liberibacter; i.e., *Ca.* L. asiaticus, *Ca*. L. africanus, and *Ca*. L. americanus [1]. In addition to graft transmission, *Ca*. L. asiaticus and *Ca*. L. americanus are transmitted by the Asian citrus psyllid *Diaphorina citri*, while *Ca*. L. africanus is transmitted by the African citrus psyllid *Trioza erytreae* [1]. While *Ca*. L. americanus and *Ca*. L. africanus are still geographically limited, *Ca*. L. asiaticus is widely distributed, being found in southeast Asia, the Indian subcontinent, the Arabian peninsula, the U.S.A., Cuba, Mexico, Jamaica, Honduras, and Brazil [1].

HLB diseased citrus plants develop a multitude of symptoms, some of which resemble zinc and copper deficiencies [1]. Visible symptoms include blotchy mottled, pale yellow and thin leaves, yellow shoots, corky veins, stunting and twig dieback. Affected fruits are small, lopsided, have aborted seeds, are acidic and bitter in taste, and ripen prematurely [1]. It has also been reported that *Ca*. L. asiaticus infection seriously affects the roots [2]. It has been observed that *Ca*. L. asiaticus infected trees are more adversely affected by extreme weathers than are healthy trees. Consequently, symptoms of stress, e.g., excessive leaf loss and premature fruit drop, occur in *Ca*. L. asiaticus infected trees. This stress intolerance is thought to result partially from a loss of fibrous root function. Recently, it was reported that HLB-diseased, four-year-old trees of Valencia sweet orange (*Citrus sinensis*) on citrumelo rootstock (*Citrus paradisi* x *Poncirus trifoliata*) showed a 31% and 38% reduction in fibrous root mass density for presymptomatic and symptomatic trees, respectively, compared to healthy trees. Similarly, HLB-diseased, three-year-old trees of Hamlin sweet orange (*C. sinensis*) on citrumelo rootstock showed a 30% and 37% reduction in fibrous root mass density for presymptomatic and symptomatic trees, respectively [3].

Anatomical aberrations in the *Ca*. L. asiaticus infected leaves compared to healthy leaves include the excessive accumulation of starch, callose depositions, phloem plugging, necrosis and collapse, the swelling of sieve elements and companion cell walls, and the disruption of chloroplast inner grana structures [1], [4]–[8]. Analyses of secondary metabolites in the *Ca*. L. asiaticus infected fruit compared to healthy fruit revealed increases in terpenes, hesperidin, naringenin, quercetin, limonin and nomilin agycones [9], [10]. Starch accumulates excessively only in aerial tissues, such as the phloem elements and vascular parenchyma in leaves and petioles, xylem parenchyma and spongy mesophyll cells and the phelloderm of stems, but it is depleted in roots [4], [6], [8]. While sucrose and fructose accumulates in both the leaf midribs and lobes, glucose accumulates only in the midribs [7]. In addition, the disruption of inner grana structures occurs only in leaf parts that are experiencing phloem plugging [5]. Rosales and Burns [11] also showed that within the same fruit, the indole-3-acetic acid concentration is higher in misshapen areas compared with those that are normal in shape. Previous studies indicate that *Ca*. L. asiaticus is distributed in bark tissue, leaf midrib, roots, and different floral and fruit parts, but not in endosperm and embryo, of infected citrus trees [12]. The leaves, stems, and roots play distinct roles in the photosynthesis and transportation of water, nutrients, etc. However, the effects of *Ca*. L. asiaticus infection on gene expression in the stems and roots remain to be elucidated despite the recent progress that has been made toward understanding the transcriptomes of leaves and fruit that are infected with *Ca*. L. asiaticus using either microarray or RNA-seq [4], [13]–[18].

Microarray analyses of infected sweet orange (*Citrus sinensis*) leaves revealed that *Ca*. L. asiaticus modulates a large cascade of molecular pathways [4], [13], [14], [17], to which some of the abovementioned phenotypes have been attributed. For example, in addition to callose deposition, the plugging of phloem elements has been linked to phloem protein 2 (PP2), which contributes to starch accumulation [4], [5], [13]. It was suggested that the excessive starch accumulation is partly due to the up-regulation of starch synthesis genes, such as ADP-glucose pyrophosphorylase (AGPase), starch synthase, granule-bound starch synthase (GBSS) and the starch debranching enzyme (SDE) [4], [13]. Recently, Fan et al. [7] reported the down-regulation of starch metabolism enzymes, such as transglucosidase and a maltose exporter. Furthermore, their work demonstrated an increase in cell wall invertase activity in HLB-affected citrus plants, which may lead to increases in sucrose and glucose. Not surprisingly, several biological processes differentially modulated in leaves during the symptomatic phase of *Ca*. L. americanus infection were similarly affected by *Ca*. L. asiaticus infection including induction of transcripts encoding zinc transporters [14], induction of transcripts encoding key enzymes involved in starch biosynthesis and repression of those related to starch breakdown, and induction of transcripts encoding P-proteins and repression of transcript encoding a salicylic acid-binding protein 3 (SABP3) [19].

Both microarray and RNA-seq techniques have been used to investigate the citrus fruit response to *Ca*. L. asiaticus infection [15], [18]. Liao and Burns [18] compared the transcriptomic changes associated with *Ca*. L. asiaticus infection in flavedo, vascular tissue, and juice vesicles from symptomatic, asymptomatic and healthy fruit based on microarray analysis. Their results indicated that many categories of metabolism including numerous genes involved in carbohydrate transport and metabolism were affected by HLB, but no category appeared to be specific to the disease. It was also suggested that mechanisms regulating development of HLB symptoms may result from the host disease response rather than being a direct consequence of carbohydrate starvation [18]. RNA-Seq was used to profile the transcriptome of citrus fruit response to *Ca*. L. asiaticus infection focusing on the peel [15]. Importantly, RNA-Seq can reveal rare and unknown transcripts and expression of genes unrepresented on the arrays, and avoid non-specific hybridization despite some drawbacks of RNA-Seq [15], [20]–[22]. It was shown that numerous pathways including those involved in photosynthesis, source-sink communication, sucrose and starch metabolism, and hormone synthesis and signaling were differentially regulated by *Ca*. L. asiaticus infection [15]. Proteomics analyses also helped us understand the host response of citrus to *Ca*. L. asiaticus infection [14], [23]. In this study, we compared the gene expression levels of stems, and roots in healthy Valencia sweet orange trees with those that were infected with *Ca*. L. asiaticus using microarrays. Microscopy analyses were also conducted to compare the stems and roots of healthy and HLB diseased citrus.

## Results

**Figure 1:**
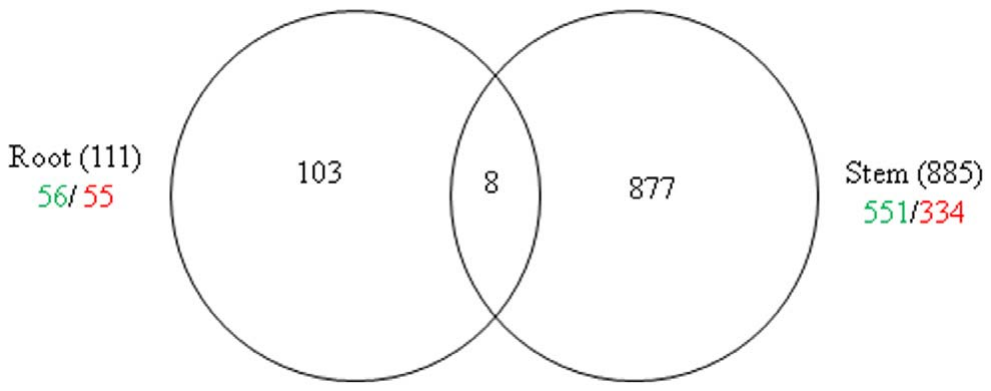
Differential regulation of genes in the stems and roots of Valencia sweet orange (*Citrus sinensis*) by *Ca*. L. asiaticus infection. Figures in parentheses indicate total numbers of genes in the stems and in the roots. The numbers of significantly up-regulated genes are shown in green, and down-regulated are shown in red. A total of 998 genes showed significantly altered expression in the stems and roots.

**Figure 2:**
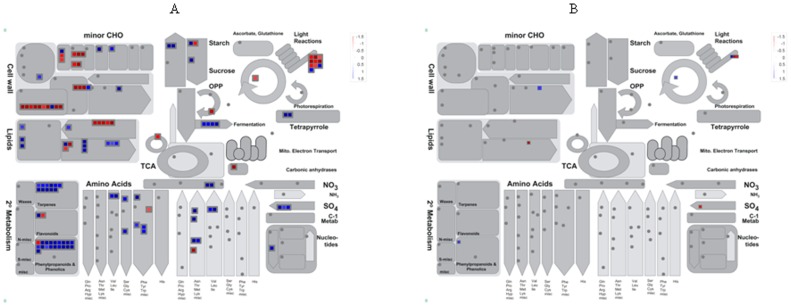
Overview of metabolic pathways that are regulated by *Ca*. L. asiaticus infection in the stems and roots of Valencia sweet orange (*Citrus sinensis*). **A** = stem and **B** = root. Genes that were significantly up-regulated following *Ca*. L. asiaticus infection are displayed in blue, and down-regulated genes are displayed in red.

**Figure 3:**
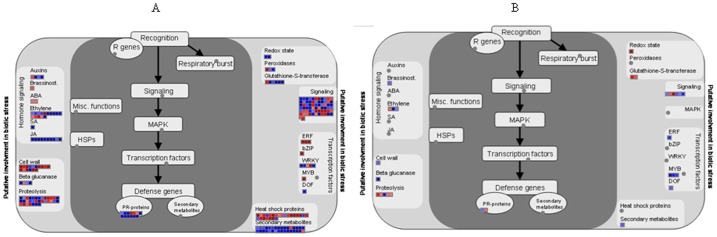
Regulation of biotic stress-related gene pathways by *Ca*. L. asiaticus infection in the stems and roots of Valencia sweet orange (*Citrus sinensis*). **A** = stem and **B** = root. Genes that were significantly up-regulated following *Ca*. L. asiaticus infection are displayed in blue, and down-regulated genes are displayed in red.

**Figure 4:**
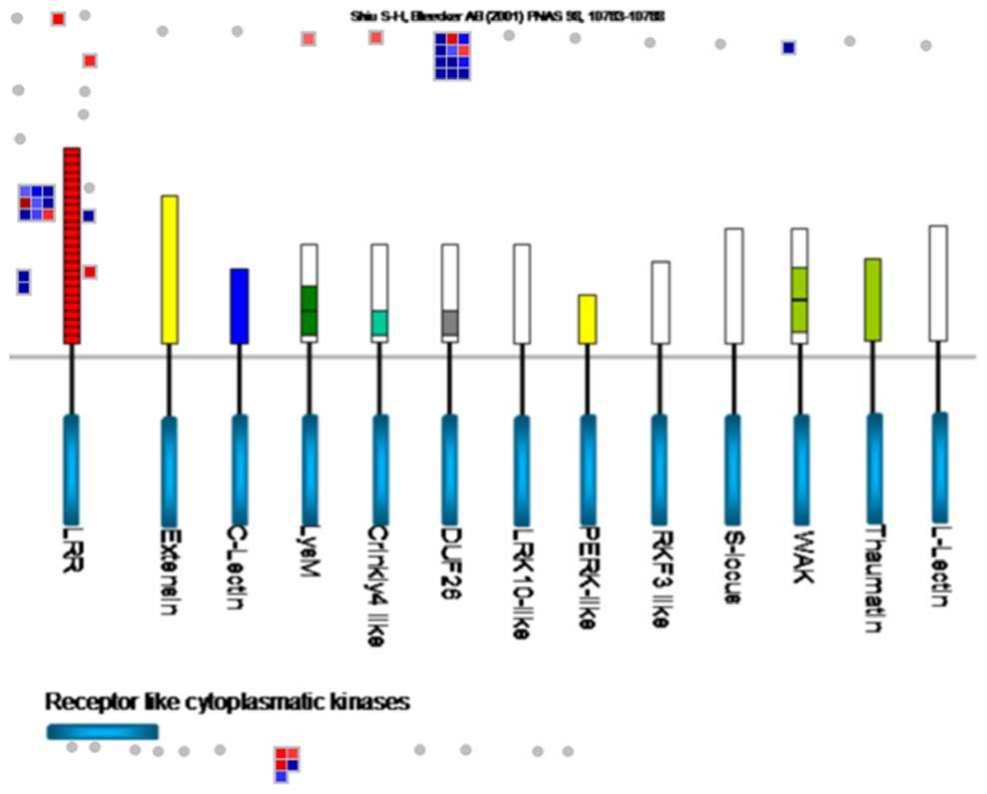
Regulation of receptor-like kinase (RLK) genes by *Ca*. L. asiaticus infection in the stems of Valencia sweet orange (*Citrus sinensis*). Genes that were significantly up-regulated following *Ca*. L. asiaticus infection are displayed in blue, and down-regulated genes are displayed in red. Abbreviations/definitions: LRR, leucine-rich repeats; Extensin, RLK with extensin motif; LysM, RLKs with lysine motif; C-lectin, RLKs with lectin-like motifs; Crinkly4-like, RLKs with crinkly4-like domains; DUF26, domain of unknown function 26; LRK 10-like, RLK gene linked to Lr10 locus; L-lectin, RLKs with lectin-binding domains; PERK-like, proline-rich extensin-like kinase; S-locus, RLK with S-domain similar to S-locus glycoproteins; RKF3-like, receptor-like kinase in flowers 3; Thaumatin, RLK-like thaumatin protein; WAK, wall-associated kinase.

To understand how *Ca*. L. asiaticus infection affects stems and roots, Affymetrix microarray analysis was conducted. After removal of probe set-related redundancies, and at a P value of <0.05 and a log_2_ fold change (LFC) of ≥1.00 or ≤−1.00 as cutoff thresholds, a total of 988 genes showed significantly altered expression, of which 885 were in the stems, and 111 in the roots. Of these, 551 and 56 were up-regulated, while 334 and 55 were down-regulated in the stem and root samples, respectively (Fig. 1). Dramatic differences in the transcriptional responses were observed between the citrus stems and roots to *Ca*. L. asiaticus infection, with only 8 genes affected in both the roots and stems. For the identification of the processes and genes that were affected, the data was analyzed using the MapMan gene ontology system [24]. We found that the affected genes are involved in diverse cellular functions (Fig. 2, Fig. S1, Fig. S2), including carbohydrate metabolism (Fig. S3), cell wall biogenesis (Fig. 2), biotic and abiotic stress responses (Fig. 3), signaling and transcriptional factors (Fig. 3, Fig. 4, Fig. S2, Fig. S4), secondary metabolism (Fig. S5), phenylpropanoid pathway (Fig. S6), transportation, cell organization, protein modification and degradation, development, hormone signaling, metal handling, redox, and enzymatic activities (Fig. S1, Fig. S2). The proportions and types of genes that belonged to the various functional groups were different between roots and stems, with the most categories altered in the stems compared with the roots. The low similarities between the stem and root expression profiles indicate that *Ca*. L. asiaticus infection affects the stems, and roots differently. Based on previous knowledge regarding the responses of citrus plants to *Ca*. L. asiaticus infection, we focused further analyses on seven gene categories that are presumed to significantly contribute to the HLB disease symptoms caused by *Ca*. L. asiaticus infection.

### Starch and Sugar Metabolism

The induction of starch accumulation by *Ca*. L. asiaticus infection has been found to be in the leaves followed by stems but not in the roots [4], [6], [7]. Sucrose and glucose accumulation by *Ca*. L. asiaticus infection has also been found in the leaves [4], [7]. Therefore, we compared the expression of carbohydrate metabolism genes in the stems and the roots. In conformity to the above, the expression of the genes encoding enzymes and proteins that are involved in both major and minor carbohydrate metabolism was only affected in the stems, but not in the roots (Fig. 2, Fig. S3, Table 1). The transcription of genes encoding an ADP-glucose pyrophosphorylase large subunit 3 (APL3), which catalyzes the rate limiting step in starch synthesis [25], and a granule-bound starch synthase (GBSS), which initiates synthesis of starch granules, was up-regulated. Among the genes encoding starch-degrading and sucrose cleaving enzymes, the genes for alpha-amylase 1 (AMY1) and exocellular acid invertase 1 (Exinv1) were up-regulated, while those encoding beta-amylase 1 (BMY1) and a neutral invertase were down-regulated. *Ca*. L. asiaticus infection also repressed the transcription of minor carbohydrate metabolism associated genes encoding trehalose biosynthesis enzymes, trehalose-6-phosphate synthases (TPS2, TPS8, and TPS10) and two trehalose-6-phosphate phosphatases, but activated the gene for myo-inositol oxygenase (EC 1.13.99.1), which cleaves inositol to D-glucuronic acid (Fig. 2, Table 1).

**Table 1:** Differentially expressed genes related to carbohydrate metabolism and cell wall biogenesis in the stems and roots of Valencia sweet orange (*Citrus sinensis*) caused by *Ca*. L. asiaticus infection.

Accession No.	Gene description	Log_2_ fold change	
		Stem	Root
**Starch synthesis**			
DN622894	ADP-glucose pyrophosphorylase large subunit 3 (APL3)	1.96	
CB292132	Granule-bound starch synthase (GBSS)	1.82	
**Starch and sucrose degradation**			
CX046632	Exocellular acid invertase 1 (Exinv1)	2.43	
CD575166	Neutral invertase	−1.00	
DN958063	Alpha-amylase (AMY1)	2.45	
CF836730	Beta-amylase (BMY1/BMY7)	−1.17	
**Minor carbohydrate metabolism**			
CN184547	putative Myo-inisitol oxygenase	1.91	
CX046914	Raffinose synthase family protein (din10)	1.51	
CX306291	Stachyose synthase	−1.11	
CD575394	Trehalose-6-phosphate phosphatase (TPPA)	−1.31	
CX636014	Trehalose-6-phosphate phosphatase, putative	−1.64	
CX309237	Trehalose-6-phosphate synthase 2 (TPS2)	−1.21	
CF831824	Trehalose-6-phosphate synthase 8 (TPS8)	−1.63	
CX306907	Trehalose-6-phosphate synthase 10 (TPS10)	−1.83	
**Cell wall precursors**			
CK939533	UDP-glucose 4-epimerase	−1.37	
**Cell wall proteins**			
CX638719	Extensin		−1.41
CX076453	Extensin-like protein	1.22	
CF417841	Proline-rich protein (PRP4)	1.09	
CK938120	Glycine-rich RNA-binding protein	1.23	
**Cellulose synthesis**			
CF835773	Cellulose synthase-like C7 (CSLC7)	−1.90	
CV705580	Cellulose synthase catalytic subunit-like protein	−1.72	
DN618266	Cellulose synthase-like protein D4 (CSLD4)	−1.44	
CX045083	Cellulose synthase isolog	1.25	
**Cell wall modification**			
DN618428	BURP domain containing protein	2.01	
CX665316	Polygalacturonase-like protein		1.10
CF835126	Expansin-related protein 1 (EXP1)	2.03	
CK939135	Syringolide-induced protein 19-1-5	−2.67	
CX296097	putative Xyloglucan endotransglycosylase (XET)	−2.80	
CV716643	Xyloglucan endotransglycosylase 1 (XET)	−2.53	

Accession No. is a unique identifier of EST sequences from several citrus species and hybrids linked to the NCBI. LFC is the ratio of the expression level in the infected samples compared to the healthy trees. The ratio is the mean of 3 replicates. The annotation is according to the latest available BLASTx search at non-redundant protein database at the NCBI. Metabolic pathway grouping is based on the gene ontology in the MapMan program (Thimm *et al*., 2004).

### Cell Wall Metabolism

Plant cell walls are composed of layers of cellulose microfibers embedded in a matrix of pectin and hemicellulose, plus some structural proteins [26]–[28]. *Ca*. L. asiaticus infection induced differential expression of multiple genes encoding enzymes and proteins involved in the synthesis, assembly, and modification of cell wall. The infection repressed the expression of genes encoding cellulose synthase-like D4 (CSLD4) and CSLC7 that mediate synthesis of β-1,4 linkages in the hemicellulose backbones, and a cellulose synthase catalytic subunit protein (Fig. 2, Table 1) [28], [29]. Mutations in cellulose synthesis associated genes lead to reduction in growth anisotropy, and display cell and organ swelling [27]. In rice, CSLD4 has been shown to be essential for normal cell-wall biosynthesis and plant growth [30]. *Ca*. L. asiaticus infection also repressed expression of a UDP-glucose 4-epimerase encoding gene. Isoforms of UDP-glucose 4-epimerase were reported to be involved in pollen development, influence cell wall galactose content, which was correlated with shoot growth, and affect root growth by changing galactant content [31]. This is consistent with the reduced shoot and root growth of the HLB diseased trees. *Ca*. L. asiaticus infection up-regulated the transcription of genes encoding structural protein components of the cell wall such as a proline-rich protein (PRP4), a glycine-rich RNA-binding protein and an extensin-like protein. Extensin is a structural protein that is involved in cell wall assembly [32]. The infection repressed expression of two genes encoding xyloglucan endotransglycosylase (XET) [33] which has been implicated in many aspects of cell wall biosynthesis including regulating wall expansion by cutting and rejoining xyloglucan to incorporate newly synthesized XG into the wall matrix [33] and in the wall degradation needed for fruit ripening [34]. *Ca*. L. asiaticus infection up-regulated genes encoding an expansin-related protein 1 (EXP1), and a BURP domain-containing protein. Expansin is a cell wall loosening protein that induces stress relaxation and extension of cell walls. Organ specific expression of EXP1 has been reported in hybrid aspen (*Populus tremula* x *Populus tremuloides* Michx) where it was highly expressed in stem tissues such as cambium/phloem but not in roots [35], [36]. In the root, only two genes were affected in expression by *Ca*. L. asiaticus infection including down-regulation of an extension gene and up-regulation of a gene encoding a polygalacturonase-like protein which hydrolyses galacturonic acid residues in cell walls (Fig. 2, Table 1).

### Disease Resistance and Pathogenesis-related (PR) Genes

The expression of disease resistance associated genes belonging to the nucleotide binding site (NBS)- leucine-rich repeat (LRR) gene family that contain centrally located NBS, C-terminal LRR and amino-terminal TIR (Toll/interleukin-1 receptor-like) or CC (coiled-coil) domains were affected predominately in the stems compared to the roots by *Ca*. L. asiaticus infection (Table 2). In the stems, reduced transcription was observed for genes encoding a NBS-LRR-like protein cD7, a TMV N-like disease resistance protein that confers resistance to tobacco mosaic virus in tobacco [37], a putative TIR-NBS-LRR-class protein, and an inter-alpha-trypsin inhibitor heavy chain-related protein (Table 2). The genes encoding a CC-NB-LRR domain containing protein, a resistance protein candidate 2 (RGC2), and a disease resistance family protein SC0A belonging to the *Cladosporium fulvum* resistance protein Cf-9 (Hcr9) family, were however, up-regulated (Table 2). In the roots, while the expression of a gene encoding the CC-NB-LRR was repressed, expression of a gene encoding a NBS-LRR protein Hom-F was up-regulated by *Ca*. L. asiaticus infection. Also specifically in the stem, genes encoding miraculin-like proteins, which are soybean Kunitz-type trypsin inhibitors that specifically inhibit the activities of membrane-bound serine proteases [38], were up-regulated. Among PR proteins, only the transcription of a PR10-encoding gene was up-regulated in the stems but was un-affected in the roots (Table 2).

**Table 2:** Differentially expressed genes related to disease resistance and pathogenesis-related proteins in the stems and roots of Valencia sweet orange (*Citrus sinensis*) caused by *Ca*. L. asiaticus infection.

Accession No.	Gene description	Log_2_ fold change	
		Stem	Root
CX670605	PR protein from class 10	1.03	
CX674271	Pathogenesis-related protein PR10A	1.43	
DN618117	NBS-LRR-like protein cD7	−1.32	
CX674648	TMV N-like disease resistance protein	−1.64	
CX671207	Inter-alpha-trypsin inhibitor heavy chain-related	−1.04	
CB322091	Disease resistance protein (TIR-NBS-LRR class)	−1.67	
CD573651	NBS-LRR type disease resistance protein Hom-F		1.05
CX075252	CC-NB-LRR protein		−1.06
DN796858	CC-NB-LRR protein	1.21	
DN798196	Resistance protein RGC2	1.28	
CX641603	Disease resistance protein, putative	1.05	
CX287601	Disease resistance family protein, SC0A	1.99	
CK934670	Miraculin-like protein 2	2.72	
CX305265	Miraculin-like protein 2	3.38	
CX043805	Miraculin-like protein 2	3.72	
CX674833	Miraculin-like protein 2	5.37	
CX045518	Miraculin-like protein 2	3.82	
CF833881	Miraculin-like protein 3	3.39	

Accession No. is a unique identifier of EST sequences from several citrus species and hybrids linked to the NCBI. LFC is the ratio of the expression level in the infected samples compared to the healthy trees. The ratio is the mean of 3 replicates. The annotation is according to the latest available BLASTx search at non-redundant protein database at the NCBI. Metabolic pathway grouping is based on the gene ontology in the MapMan program (Thimm *et al*., 2004).

### Signaling Molecules and Receptor-like Kinases

The expression of several genes encoding receptor-like kinases (RLK) which regulate plant development and defense response was affected in the stems but not in the roots (Fig. 3, Table 3). *Ca*. L. asiaticus infection up-regulated expression of 12 plant defense genes (Fig. 3) such as homologs of *Xa21*, *Hcr2-5D*, *Cf-5* and *Cf-2.2*, and a gene encoding a systemin receptor SR160 which was reported to induce systemic defense genes in wounded tomato plants [39]. Five development related genes including genes encoding a receptor protein kinase ERECTA and a receptor-like protein kinase 1 were down-regulated in the stems by *Ca*. L. asiaticus infection (Table 3). *Ca*. L. asiaticus infection up-regulated genes encoding FERONIA (FER) in the catharanthus roseus-like gene family [26] and WAK-like kinase (WLK2) in the wall-associated kinase gene family (Table 3). Cell wall-associated kinase (WAK) and WLKs family gene products which differ from RLKs by containing a cytoplasmic serine/threonine kinase domain and an extracellular region containing epidermal growth factor-like repeats regulate cell elongation and plant development as well as pathogen and heavy-metal tolerance [40].

**Table 3:** Differentially expressed genes related to signaling in the stems and roots of Valencia sweet orange (*Citrus sinensis*) caused by *Ca*. L. asiaticus infection.

Accession No.	Gene description				Log2 fold change			
					Stem		Root	
**Crinkly like**								
CV711750	putative Protein kinase				−1.06			
**Lysine motif**								
CX293076	putative Protein kinase					−1.01		
**Wall associated kinase**								
CD574549	WAK-like kinase (WLK2)				4.88			
**Catharanthusroseus-like RLK**								
DN622543	FERONIA receptor-like kinase				1.15			
**MAP kinase**								
CX073386	MPK3				−1.44			
**Receptor like cytoplasmatic kinase**								
CN184216	protein Serine threonine kinase-like			−1.25				
CV707629	Receptor protein kinase-like protein			−1.14				
CX670980	putative Protein kinase			1.58				
CV719828	AT3G57730			1.16				
**DUF 26**								
CN185603	hypothetical protein F19K19.4			−1.39				
CN192263	AT1G01540			−1.13				
DN625179	Receptor protein kinase-like protein			1.70				
CV710863	putative Receptor-like protein kinase 4 RLK4			1.65				
CV706414	Receptor protein kinase			1.24				
CX674957	hypothetical protein F20P5.3			1.93				
CX640171	putative Serine threonine kinase			1.60				
CX295060	putative Serine threonine kinase			1.07				
DN617645	Receptor protein kinase			2.43				
**Leucine rich repeat**								
CV707041	probable Receptor kinase		−1.28					
CX675277	putative Receptor protein kinase, ERECTA		−1.57					
CX043848	putative Receptor protein kinase, ERECTA		−1.37					
CX671538	Hcr2-5D		2.37					
CX671347	Cf-2.2		1.38					
CX641603	Disease resistance protein, putative		1.05					
DN624314	Disease resistance protein (cf-5)		1.84					
CX674240	hypothetical protein		1.98					
CX669184	Leucine-rich repeat receptor-like kinase		1.62					
CX072406	OSJNBa0020J04.8		−1.19					
CK937178	OSJNBa0021F22.13		2.65					
CX674269	putative Protein kinase Xa21		1.10					
CX669563	putative Receptor kinase		2.27					
CX045546	putative Receptor-like protein kinase		1.03					
DT214563	putative Systemin receptor SR160		2.51					
DN623196	Receptor protein kinase-like protein		2.02					
CX669844	Receptor-like protein kinase 1		−1.18					
**Calcium signaling**								
DN622169	Avr9/Cf-9 rapidly elicited protein		−1.16					
CK935615	Calcium-binding allergen Ole e 8		−1.59					
CN188862	Calcium-binding EF-hand family protein-like		−1.91					
CK701540	Calcium-binding pollen allergen, Polcalcin						1.13	
CX047037	EF-hand Ca^2+^-binding protein CCD1		−1.49					
CX306727	39 kDa EF-Hand containing protein		−1.37					
DN795254	putative Calmodulin-binding protein		−1.63					
CK935677	putative Calmodulin-related protein		−1.62					
DN799085	putative Calreticulin		1.43					
CK933894	unnamed protein product		−1.07					
**Sugar and nutrient physiology**								
CX294586	Glutamate receptor family protein (GLR3.3)		2.63					
CF837692	Ligand gated channel-like protein		1.10					
CV711517	phosphate-induced protein 1(PHI-1)-like protein		1.60					
CF838115	Phosphate-responsive protein, putative		1.15					
CX292843	Photoassimilate-responsive protein precursor PAR-1a		−1.70				2.12	
CV710376	Ionotropic glutamate receptor homolog GLR4						1.03	
CX288476	putative Ligand-gated ion channel subunit	1.50						

Accession No. is a unique identifier of EST sequences from several citrus species and hybrids linked to the NCBI. LFC is the ratio of the expression level in the infected samples compared to the healthy trees. The ratio is the mean of 3 replicates. The annotation is according to the latest available BLASTx search at non-redundant protein database at the NCBI. Metabolic pathway grouping is based on the gene ontology in the MapMan program (Thimm *et al*., 2004).

The expression of genes that are involved in transducing Ca^2+^ signals was altered by *Ca*. L. asiaticus infection. In the stems, expression of eight genes was repressed including genes encoding a calcium-dependent protein kinase Avr9/Cf-9 rapidly elicited protein (ACRE), a calcium-binding allergen Ole e 8, a calmodulin-binding protein, an EF-hand Ca2+-binding protein CCD1, and a 39 kDa EF-hand-containing protein but only one gene encoding calreticulin was up-regulated (Table 3). In the roots, only the transcription of a gene for calcium binding pollen allergen, polcalcin, was activated (Table 3). Among genes whose products are involved in sugar sensing and nutrient physiology, genes encoding a glutamate receptor (GLR) family protein GLR3.3, a phosphate-responsive protein, and a phosphate-induced 1 (PHI-1)-like protein were up-regulated in the stems (Table 3). The gene encoding a photoassimilate-responsive protein PAR-1a was repressed in stems, but up-regulated in the roots (Table 3). *Ca*. L. asiaticus infection also up-regulated the expression of a gene encoding an ionotropic glutamate receptor homolog GLR4 (Table 3).

### Transcription Factors

*Ca*. L. asiaticus infection up-regulated the expression of a gene encoding an Arabidopsis response regulator 1 (ARR1) homolog but repressed the expression of a gene encoding a lateral organ boundaries domain protein 38 (LOB38) in the roots (Fig. 4, Fig. S4, Table 4). LOBs perform numerous developmental functions and are known to be expressed in boundaries of the shoot and at the base of secondary roots [41], [42]. In the stems, expression of four genes encoding AUX/IAA family proteins was repressed by *Ca*. L. asiaticus infection (Table 4). Meantime, expression of an auxin response factor (ARF10) gene was also repressed (Table 4). Genes encoding basic helix-loop-helix (bHLH) family proteins showed both patterns of regulation (4 repressed and 2 up-regulated). Noticeably, the transcription of a gene encoding a homeobox-leucine zipper protein was repressed in the stems but was up-regulated in the root (Table 4). *Ca*. L. asiaticus infection repressed the expression of three genes but induced one gene belonging to the APETALA2/Ethylene-responsive element binding family in the stems and in the roots respectively (Table 4). Expression of two MYB domain TF family genes including a tuber-specific and sucrose-responsive element binding factor gene was repressed in stems, whereas in the roots three genes in the same family including MYB68 were up-regulated (Table 4). Importantly, its homolog AtMYB68 is a root growth specific regulator, impacting overall plant development under unfavorable conditions [43]. MYB68 is a negative regulator of lignin deposition in Arabidopsis [43], and its down-regulation may affect lignification in *Ca*. L. asiaticus infected citrus. *Ca*. L. asiaticus infection only affected the expression of genes encoding proteins containing WRKY domain in the stems, wherein, it increased the expression of WRKY4 and WRKY23 but repressed that of WRKY30 (Table 4). Overexpression of WRKY4 enhanced susceptibility of Arabidopsis plants to *P. syringae* and suppressed PR1 gene expression [44]. The effect of up-regulation of WRKY4 in *Ca*. L. asiaticus infected citrus remains to be addressed.

**Table 4:** Differentially expressed genes related to transcriptional factors in the stems and roots of Valencia sweet orange (*Citrus sinensis*) caused by *Ca*. L. asiaticus infection.

Accession No.	Gene description	Log2 fold change					
		Stem			Root		
**AP2/EREBP, APETALA2/Ethylene-responsive element binding protein family**							
CF836437	putative AP2 domain transcription factor				1.27		
CN185220	Transcription factor TINY, putative		−2.56				
CF831712	AP2 domain transcription factor-like protein		−1.80				
CK939963	CaCBF1B		−2.12				
**ARF, Auxin Response Factor family**							
CX294853	Auxin response factor 10		−1.17				
**Arabidopsis response regulator (ARR)**							
BQ623949	Response regulator1 (ARR1)				1.19		
**AS2, Lateral Organ Boundaries Gene Family**							
CF834266	LOB domain protein 38				−1.59		
**Aux/IAA family**							
CD574798	AUX/IAA protein		−2.21				
DR404247	AUX IAA protein		−1.18				
CV704184	AUX IAA protein		−1.07				
CV708756	Auxin-regulated protein		−1.46				
**bHLH, Basic Helix-Loop-Helix family**							
CX045057	Basic helix-loop-helix (bHLH) family protein		2.63				
CK932760	bHLH transcription factor, putative		−1.04				
CX676159	Helix-loop-helix DNA-binding protein-like		−1.60				
CF506528	putative bHLH transcription factor		−1.60				
CX671833	putative Transcription factor RAU1		1.39				
CX044856	unknown protein		−1.11				
**bZIP transcription factor family**							
CF507428	bZIP transcription factor		−3.04				
**C2C2(Zn) CO-like, Constans-like zinc finger family**							
CX048448	Zinc finger (B-box type) family protein		−1.62				
**C2C2(Zn) DOF zinc finger family**							
DN798578	unknown protein		1.49				
CX640084	unknown protein					1.10	
**C2C2(Zn) GATA transcription factor family**							
CX049639	putative protein		−1.08				
**Zinc finger family**							
DN622949	Zinc finger protein, ZPT2-12		−1.46				
DN797852	Zinc finger protein, ZPT2-12		−1.52				
CK939958	Zinc finger (CCCH-type) family protein		−1.56				
**GRAS transcription factor family**							
CN186577	Scarecrow gene regulator		−1.62				
CB291917	Scarecrow transcription factor family protein		−1.25				
CX670106	Scarecrow-like transcription factor 14 (SCL14)		−1.14				
CV710984	AP2 SCARECROW-like protein		1.01				
**HB, Homeobox transcription factor family**							
DN798602	Homeobox leucine zipper protein		−1.42		1.21		
CV704865	Homeobox 2 protein		1.06				
**MYB domain transcription factor family**							
CX307046	MYB-like DNA-binding domain protein				1.68		
CK936024	MYB family transcription factor (MYB68)				1.30		
CF834992	MYB-related transcription factor		−1.42		1.20		
CF506570	Tuber-specific and sucrose-responsive element binding factor		−2.69				
**WRKY domain transcription factor family**							
CK939682	putative WRKY transcription factor 30 (WRKY30)		−1.91				
AJ489051	putative WRKY4 transcription factor		3.16				
CX050828	putative WRKY-type DNA binding protein 23 (WRKY23)		1.51				
CX643787	WRKY transcription factor		1.32				

Accession No. is a unique identifier of EST sequences from several citrus species and hybrids linked to the NCBI. LFC is the ratio of the expression level in the infected samples compared to the healthy trees. The ratio is the mean of 3 replicates. The annotation is according to the latest available BLASTx search at non-redundant protein database at the NCBI. Metabolic pathway grouping is based on the gene ontology in the MapMan program (Thimm *et al*., 2004).

Zinc finger-containing transcriptional factors that are involved in plant developmental and defense showed both patterns of regulation in the stems (Table 4). Expression of the following genes were repressed in the stem: ZPT2-12, a member of the EPF-type zinc fingers that are involved in flower development and plant defense against pathogens [45]; a gene encoding a CCCH-type zinc finger family protein; and a gene of the C2C2(Zn)-GATA family that has been implicated in light-responsive gene transcription in *Arabidopsis* [46], and a gene of the C2C2(Zn)-CO-like family that regulates circadian clock-responsive genes [47]. *Ca*. L. asiaticus infection induced the expression of a gene of C2C2(Zn)-DOF family that are involved in the regulation of carbohydrate metabolism and plant defense [48], [49] in the stems and in the roots, respectively. With the exception of a gene encoding an AP2 SCARECROW (SCR)-like (SCL) protein, expression of three genes belonging to the GRAS transcription factor family was repressed in the stems, including SCL14 and a scarecrow gene regulator highly related to chitin-inducible gibberellin-responsive proteins (Table 4). SCR and SCL proteins are involved in GA-mediated regulation of plant growth [50]. Arabidopsis mutants of SCR showed aberrant root growth and impaired development in aerial plant organs such as hypocotyl, inflorescence stems and shoot axial organs [51], [52]. Some of these symptoms are similar to those induced by *Ca*. L. asiaticus.

### Transportation

The expression of numerous genes that are involved in transportation of carbohydrates, drugs, water, oligosaccharides, and amino acids was altered by *Ca*. L. asiaticus infection in the stems (Table 5). Among the carbohydrate transporters, *Ca*. L. asiaticus infection repressed the expression of a sugar transporter gene CiSUT1 but increased the transcription of genes encoding a hexose transporter 6 (HEX6) and a sorbitol transporter in the stems. In the roots, only the transcription of a sorbitol transporter gene was repressed (Table 5). Expression of three genes encoding amino acid transporters was up-regulated whereas one was down-regulated in the stems. Only one gene whose expression was up-regulated, encoding a putative amino acid transporter highly related (e-value, 4e-41) to the amino acid/polyamine transporter II of *Medicago truncatula*, was identified in the roots. *Ca*. L. asiaticus is auxotrophic for at least five amino acids [53]. It remains to be determined whether the up-regulation of such transporter genes results from the metabolic incapacity on certain amino acids of *Ca*. L. asiaticus.

**Table 5:** Differentially expressed genes related to transportation in the stems and roots of Valencia sweet orange (*Citrus sinensis*) caused by *Ca*. L. asiaticus infection.

Accession No.	Gene description	Log_2_ fold change	
		Stem	Root
**Sugar transporter**			
CK936504	putative Sorbitol transporter		−1.41
DN958552	Sorbitol transporter	1.25	
CB292174	Hexose transporter, putative	1.28	
CX665499	Hexose transport protein HEX6	1.28	
CK936487	Citrus sucrose transporter 1 (CiSUT1)	−1.16	
**Amino acid transporters**			
CF418599	Amino acid transporter family protein		1.26
CV713475	putative Cationic amino acid transporter	−1.02	
CX298643	Amino acid transporter family protein	1.16	
CF832082	Cationic amino acid transporter 5 (CAT5)	1.62	
CK935365	putative Amino acid transport protein	1.45	
**Metabolite transporters**			
CK939426	Mitochondrial substrate carrier family protein	−2.05	
**Major Intrinsic Proteins (NIP, PIP, SIP)**			
CX638658	Major intrinsic protein	−2.47	
DN958192	NOD26-like intrinsic protein 6;1	2.71	
CX644460	putative Aquaporin (PIP1-1)	−1.50	
CV719411	Plasma membrane aquaporin (PIP1A)	−1.03	
AU300362	Putative aquaporin (PIP1-3)	−1.38	
CK933592	Small basic membrane integral protein 1A (SIP1A)	1.68	
**Metal transport**			
CX297247	Metal tolerance protein B1 (MTPB1)	−1.02	
DN619440	putative Zinc transporter	1.48	
CB290596	Zinc transporter protein, ZIP1	3.76	
**Peptides and nucleotide transport**			
CN189143	putative Proton-dependent oligopeptide transport (POT) family	1.51	
BQ622927	Yellow stripe like 5 (YSL5)	1.51	
DN622714	Nitrate transporter 1	1.57	
CX305691	Nitrate transporter NRT1-2	3.72	
**Phosphate and potasium transport**			
CX044970	Phosphate transporter 3	1.29	
CX047721	Potassium channel tetramerisation domain-containing protein	−1.08	
**Vesicle transport and secretory pathways**			
CX293833	putative Coatomer protein complex, subunit beta 2	1.74	
CV708721	unknown protein	−1.66	
CX295788	CAO	−1.18	
CV885826	VAMP family protein	−1.03	
CF653150	VAMP family protein	−1.09	
CF837201	putative RNA-binding protein	−1.49	
**ABC transporters and multidrug resistance systems**			
CB292087	putative ABC transporter family protein	3.11	−1.80
CX668058	P-glycoprotein-like protein	1.28	
CX293439	putative MRP-like ABC transporter	1.11	
**Miscellaneous**			
CX671045	putative protein	−1.04	
CV709319	Purine permease 1 (PUP1)	1.37	
CV707033	Urea transporter DUR3	1.61	
CF828901	putative MATE efflux protein family protein	1.18	
CX674953	MATE efflux family protein	1.31	
CV706445	Chloride channel-like (CLC) protein, putative	1.22	
CX670490	Nucleobase ascorbate transporter 12 (NAT12)		1.40

Accession No. is a unique identifier of EST sequences from several citrus species and hybrids linked to the NCBI. LFC is the ratio of the expression level in the infected samples compared to the healthy trees. The ratio is the mean of 3 replicates. The annotation is according to the latest available BLASTx search at non-redundant protein database at the NCBI. Metabolic pathway grouping is based on the gene ontology in the MapMan program (Thimm *et al*., 2004).

Among metal transporter genes, *Ca*. L. asiaticus infection activated the expression of genes encoding two zinc transporters (such as ZIP1), but repressed that of a metal tolerance protein B1 (MTPB1) (Table 5). In addition, expression of genes encoding transporters that move peptides, oligopeptides, and ions across membranes, including a POT family gene, NRT1 and YSL5, was up-regulated in the stems but not in the roots (Table 5). Expression of genes whose products perform various functions such as PUP1 (purine permease 1) which is involved in the uptake and transportation of cytokinins [54]; urea transporter DUR3 involved in acquisition, transportation, and utilization of urea [55]; and a chloride channel-like (CLC) protein which moves chloride ions across membranes, and two multidrug and toxic compound extrusion (MATE) efflux family proteins was also up-regulated (Table 5). The expression of a gene encoding a nucleobase ascorbate transporter 12 (NAT12) was up-regulated in the roots (Table 5).

### Secondary Metabolic Pathway

The expression of genes encoding proteins and enzymes that synthesize flavonoids, isoprenoids, phenylpropanoids and lignin was mostly up-regulated by *Ca*. L. asiaticus infection (Table 6). Only the expression of one gene encoding a naringenin-chalcone synthase 4 (CHS4) was repressed in the stems. *Ca*. L. asiaticus infection induced the transcription of 10 genes in the isoprenoid metabolic pathway such as genes encoding a 1-deoxyxylulose 5-phosphate synthase which catalyses the rate-limiting step in the production of isopentenyl diphosphate, the main precursor of all isoprenoids, a beta-amyrin synthase, a geranyl diphosphate synthase small subunit, a homogentisate geranylgeranyl transferase, two linalool synthases, and a gamma-terpinene synthase (Table 6). Expression of genes involved in the phenylpropanoid pathway were similarly up-regulated including genes encoding phenylalanine ammonia lyase (PAL; EC 4.3.1.5), a key enzyme that converts P-phenylalanine to trans-cinnamic acid, a precursor for various phenylpropanoids [56], a hydroxycinnamoyl transferase (HCT; EC 2.3.1.133), which catalyzes the conversion of *p*-coumaroyl CoA or cafeoyl CoA with shikimic acid to p-coumaroyl shikimate or caffeoyl shikimate; as well as a 10-hydroxygeraniol oxidoreductase, a caffeic acid O-methyltransferase II, the 4-coumarate-CoA ligase-like protein and catechol O-methyltransferase (Fig. S6, Table 6). The expression of only one gene encoding a transferase family protein closely related to anthranilate N-benzoyltransferase involved in phenylpropanoids pathway was induced in the roots (Table 6).

**Table 6:** Differentially expressed genes related to secondary metabolism in the stems and roots of Valencia sweet orange (*Citrus sinensis*) caused by *Ca*. L. asiaticus infection.

Accession No.	Gene description	Log2 fold change	
		Stem	Root
**Flavonoids**			
CX672036	putative Flavanone 3-hydroxylase	2.21	
CX045954	Naringenin-chalcone synthase 4	−1.14	
**Isoprenoids**			
CX302245	1-deoxyxylulose 5-phosphate synthase	1.15	
CF838068	Beta-amyrin synthase	1.58	
CX290062	Geranyl diphosphate synthase small subunit	2.70	
CX667086	GGPP synthase	1.28	
CX043706	HMG-CoA synthase 2	1.22	
CX665915	Homogentisate geranylgeranyl transferase	2.14	
CV886253	Linalool synthase	2.51	
CV885575	Linalool synthase	3.41	
CX045048	Gamma-terpinene synthase	3.75	
CX671596	Acetyltranferase-like protein	1.29	
CK937255	Acetyltranferase-like protein	−1.23	
**Phenylpropanoids/lignin biosynthesis**			
CX671596	Acetyltranferase-like protein	1.29	
CX044256	Hydroxycinnamoyl transferase	2.12	
CF830793	Transferase family protein	1.47	
CX663894	Transferase family protein		1.11
CV884611	4-coumarate-CoA ligase-like protein	1.33	
CV705977	4-coumarate-CoA ligase-like protein	1.14	
CB291954	10-hydroxygeraniol oxidoreductase	2.08	
DN622570	putative Orcinol O-methyltransferase	3.86	
CX043719	Caffeic acid O-methyltransferase II	1.73	
CX303448	Catechol O-methyltransferase	1.34	
CX670983	Catechol O-methyltransferase	2.53	
CX302017	O-methyltransferase	1.34	
CX643181	Phenylalanine ammonia-lyase 2 (PAL2)	3.62	
CF835217	Phenylalanine-ammonia lyase	2.55	
**Amino acid synthesis**			
DN620167	Prephenate dehydrogenase family protein	1.24	
CF834506	Prephenate dehydratase family protein	1.10	
CV706063	3-deoxy-D-arabino-heptulosonate 7-phosphate synthase	1.75	

Accession No. is a unique identifier of EST sequences from several citrus species and hybrids linked to the NCBI. LFC is the ratio of the expression level in the infected samples compared to the healthy trees. The ratio is the mean of 3 replicates. The annotation is according to the latest available BLASTx search at non-redundant protein database at the NCBI. Metabolic pathway grouping is based on the gene ontology in the MapMan program (Thimm *et al*., 2004).

### Validation of Differentially Expressed Genes by Quantitative Reverse Transcription PCR (qRT-PCR)

To confirm the validity of the microarray experiment, qRT-PCR assays were performed. Seven genes encoding the SBIP1A, PRP4, CAT5, PAR-1a, GLR4, the tuber-specific and sucrose-responsive element binding factor (TSF), and ZIP1 were chosen for this confirmation (Table 6). These genes were chosen because they showed distinct patterns of expression. Common patterns of expression changes were observed by the two methods, indicating the reliability of the microarray data (Table 7).

**Table 7:** Comparison of expression of selected genes in the stems and roots of Valencia sweet orange (*Citrus sinensis*) to *Ca*. L. asiaticus infection based on quantitative reverse transcription-PCR and microarray analyses.

	Gene product description	Microarray		qRT-PCR^a^	
		Stems	Roots	Stems	Roots
CK933592	Small basic membrane integral protein	1.68		3.65	
CF417841	Proline-rich protein PRP4	1.09		1.40	
CF832082	Cationic amino acid transporter 5	1.62		2.13	
CX292843	Photoassimilate-responsive protein PAR-1a		2.20		4.96
CV710376	Ionotropic glutamate receptor homolog GLR4		1.03		2.42
CF506570	Tuber-specific and sucrose-responsive element binding factor	−2.69		−2.93	
CB290596	Zinc transporter protein ZIP1	3.75		2.56	

a Fold change value calculated according to the method of Livak and Schmittgen (2001). The following primers designed using the PrimerQuest^SM^ program (Integrated DNA Technologies, Inc) were used, CK933592: TGCAGTGTTGACATCTCTGTGGGT/ACTGGTAACAGGGCTTCAACTCCA;

CF417841: ATCAGGCACTCCATGTCCAGCTTA/GTATAAGCAGCGGTTGAAGCAGCA; CF832082: TGTCGCGCTTCTTGTGAGGAGATA/TCCCAAGAACCAAAGAGGAACGGT; CX292843: TATGCGAAGCACAAGGGAAAGGTG/AAAGTTCCGGTTATGTGGCACCGGC;

CF506570: ACCAGGCTTGTCGAACGATATGGA/TGCATGATTTCCCAGACCTTCCCT;

CV710376: TGCTTGGCTTCAGGTCAGGGAATA/AGCCTCCGAACGTGTAAGTGTTGA;

CB290596: AAGGGATATTCAACGCAGCAGCAG/ACAAGGACATGCAACCAGCTCCTA.

### Microscopy Analyses of Stems and Roots Infected by *Ca*. L. asiaticus

**Figure 5:**
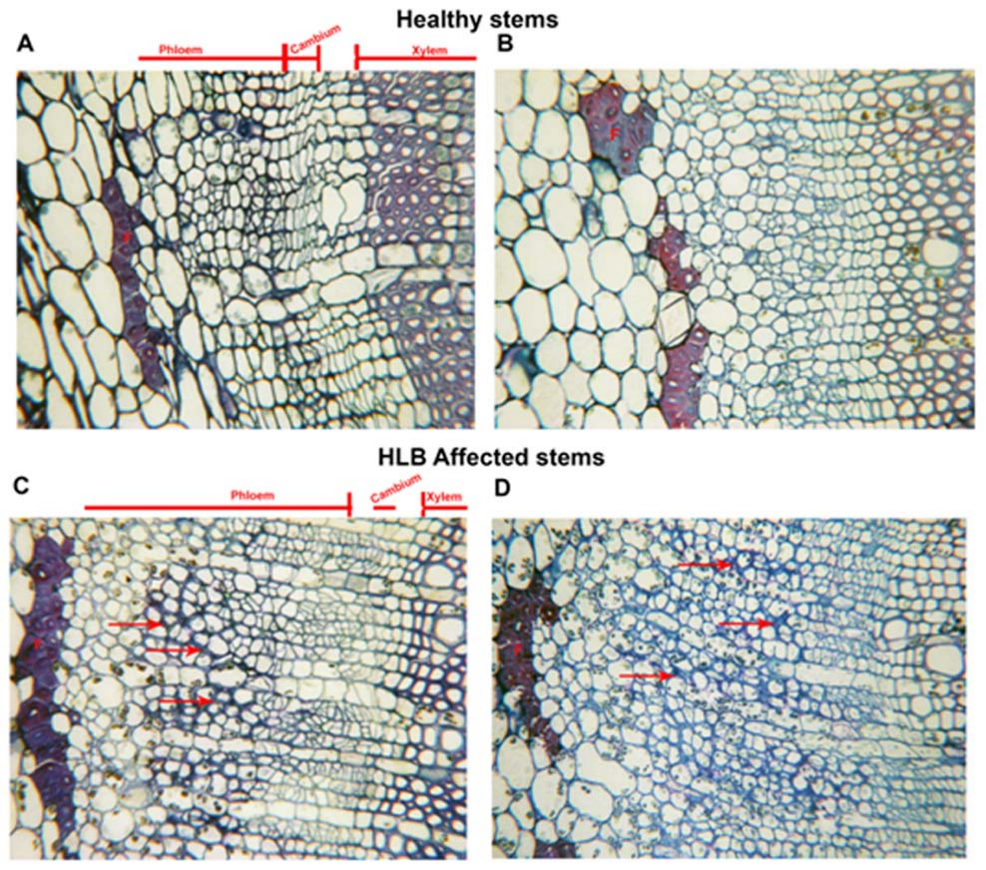
Microscopic analyses of stems of healthy and HLB affected Valencia sweet orange. **A, B.** Light microscopy of cross-sections of healthy young stems showing the phloem, cambium and xylem cells. F-Phloem fibers. **C,D** Cross section of HLB affected young stems showing greater thickness of the phloem layer compared to the healthy. Arrows point to thickened cell walls.

**Figure 6:**
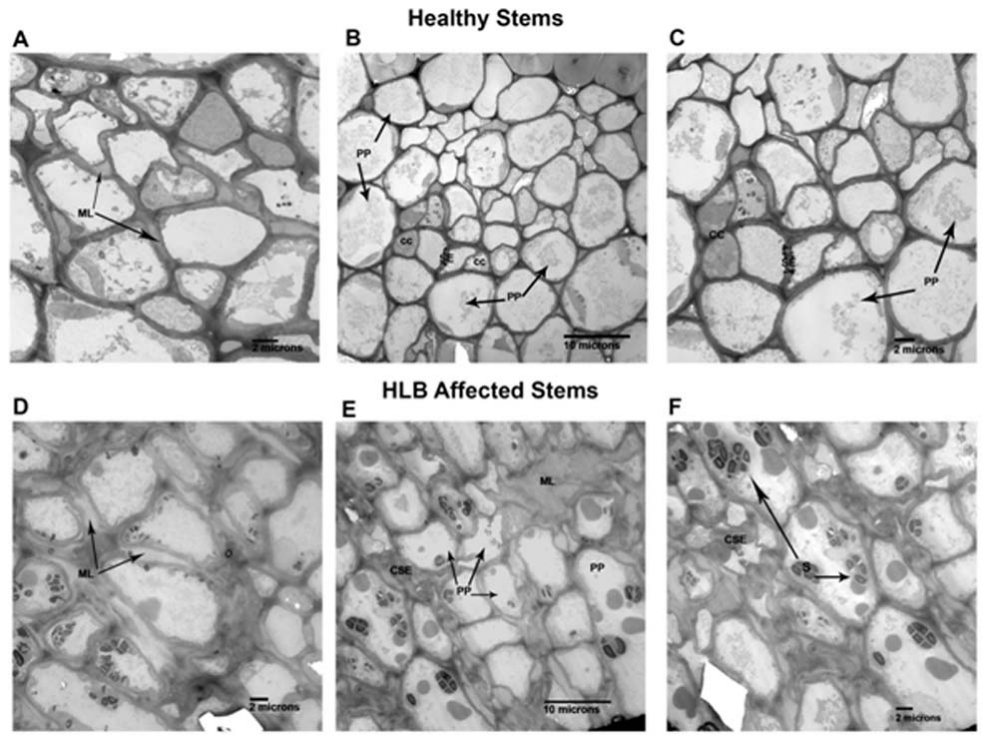
Microscopic analyses of stems of healthy and HLB affected Valencia sweet orange. **A, B, C** are electron microscopy of cross- sections of healthy young stems. Arrows in **A** point to normal middle lamella found in healthy stem phloem**. B** is a lower magnification of healthy phloem showing normal companion cells (CC) on either side of sieve elements (SE) which are surrounded by phloem parenchyma cells (PP). **C** is an enlargement of the sieve element (SE) area pictured in **B**. **D, E, F** are cross-sections of HLB affected young stems. Arrows in **D** point to swollen middle lamella comparable to dark blue stained walls shown in **Fig. 5. C, D**. **E** shows phloem in affected stem comparable in magnification to **B** showing collapsed sieve element (CSE) surrounded by normal looking phloem parenchyma (PP) some of which contain starch (S). In upper right of micrograph is area showing swollen middle lamella (ML). **F** is an enlargement of area in lower left corner of **E**, showing collapsed sieve element (CSE) and parenchyma cells containing starch (S).

Light microscopy analyses of the stems of healthy and *Ca*. L. asiaticus infected plants were shown in Fig. 5. Phloem fibers were shown on the outer boundary of the phloem layer, with cambium and xylem on the inner boundary (Fig. 5). The difference between the stems from the healthy and *Ca*. L. asiaticus infected plants observed with light microscope was the increased amount of cell layers in the phloem and the increased thickness of the cell walls of some of the cells in this area (arrows). When observed at higher magnification (TEM), the following were observed for the stems of *Ca*. L. asiaticus infected plants: a swelling in the middle lamella (Fig. 5 D, E) in some areas and, in others, the increase in thickness and collapse of cell walls (Fig. 5 E, F) that appeared to be those of the sieve elements (SE) and companion cells (CC). This collapse observed in the stem was similar to, but not as severe as, that reported in petioles and mid-ribs of leaves affected by HLB. There was also typically more starch (S) in the HLB affected tissues (Fig. 5 C, D, Fig. 6 E, F) compared to the healthy.

**Figure 7:**
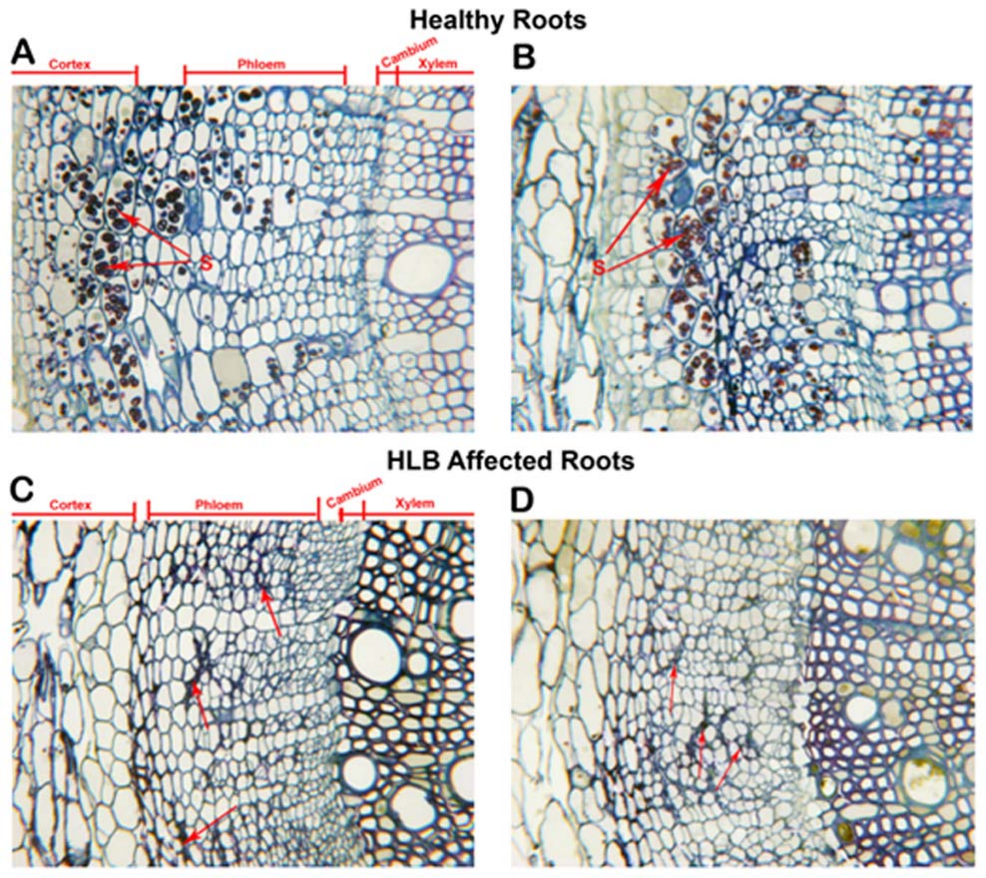
Microscopic analyses of roots of healthy and HLB affected Valencia sweet orange on citrumelo rootstock (*Citrus paradisi* x *Poncirus trifoliata*). **A, B.** Light microscopy of cross-sections of healthy fibrous roots showing secondary thickening. The cortex and some phloem parenchyma cells are shown containing starch (S). The cortex, phloem, cambium and xylem areas are delineated by lines. **C, D** are cross-sections of HLB affected roots. Note that the phloem layers of these roots are comparable in thickness to those of healthy above. The arrows point to collapse, thickened cell walls.

**Figure 8:**
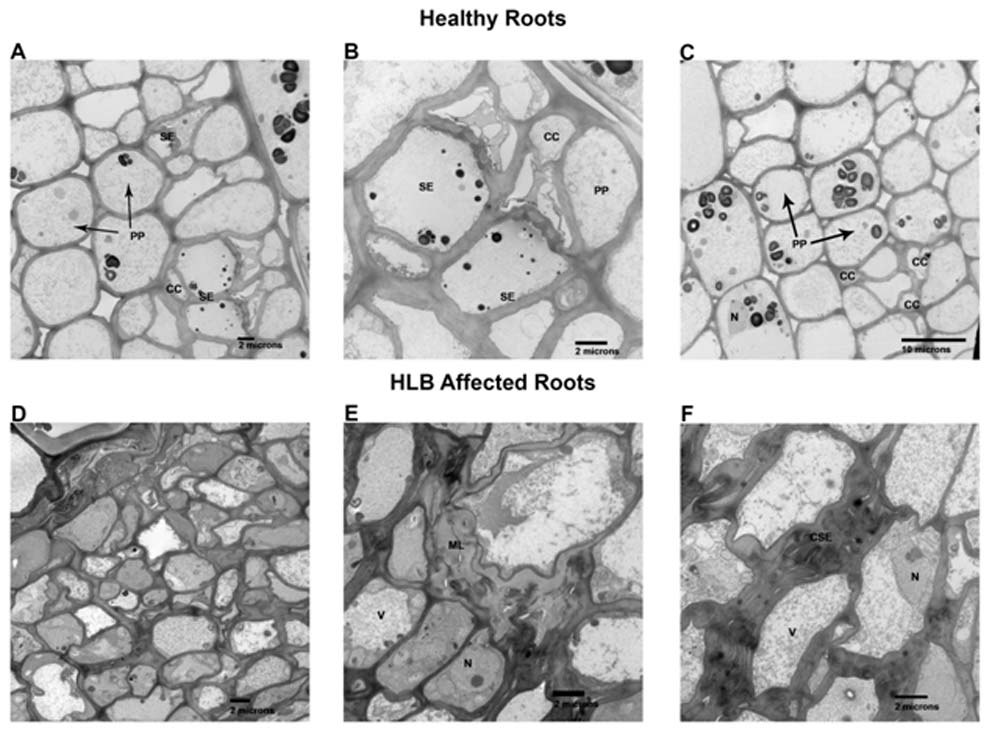
Microscopic analyses of roots of healthy and HLB affected Valencia sweet orange on citrumelo rootstock (*Citrus paradisi* x *Poncirus trifoliata*). **A–D.** Electron microscopy of healthy fibrous roots. **A & C** are relative low magnifications showing size and shape of normal phloem parenchyma (PP), sieve elements (SE) and companion cells (CC**). B** shows a higher magnification of same areas enhancing the sieve element areas. **D–F** are HLB affected roots. **D** is a low magnification comparable to **A & C** above showing the enriched cytoplasmic contents of these cells compared to the healthy above. **E** shows enlarged middle lamellas (ML). **F** shows the collapsed sieve elements (CSE). N, prominent nucleus and V, vacuole.

The comparison of healthy and HLB affected roots did not reveal as marked a contrast as in the stem (Fig. 7). The major differences included more starch (S) found in the roots of healthy plants (Fig. 7 A, B) compared to the HLB affected root (Fig. 8 C, D). There was not an increase in the development of phloem cells in the HLB roots as there was in the HLB stem tissue (Fig. 6). At the light microscope level, the collapse and thickening of cell walls of HLB affected roots (Fig. 7 C, D arrows) were not as severe as in the stems (Fig. 5). At the higher magnification (TEM), thickening of the middle lamella (ML) (Fig. 4, E) and thickening and collapse of the sieve elements (CSE) (Fig. 4 F) were also observed in the HLB affected roots. Another difference observed was the apparent greater density of the cytoplasm (Fig. 8 D) in the HLB affected phloem compared to that in the cells of the healthy plant (Fig. 8 A, C). Nuclei (N) and other organelles (Fig. 8 E, F) in the phloem parenchyma (PP) and companion cells (CC) in HLB affected roots were more prominent than in the healthy roots.

## Discussion

Dramatic differences in the transcriptional responses were observed between the citrus stems and roots to *Ca*. L. asiaticus infection (Fig. 1). Overall, 885, and 111 genes were regulated in the stems, and roots, respectively, by the infection, with only 8 genes overlapping (Fig. 1). In contrast to the low similarity between the expression profiles of the stems and roots, the expression profile of the stem to *Ca*. L. asiaticus infection is very similar to the leaf [4], [13]–[15], [17], [57]. For example, when we compared our data with the leaf expression profile [4], 291 genes were regulated in both the stems and the leaves compared to the 8 genes shared between the stems and roots (Fig. 1). The low similarity among the stem and root expression profiles might result from that *Ca*. L. asiaticus affects the stems and roots differently due to the distinct functions of the stems and roots, and the different physiological changes caused by the pathogen. Depletion of starch in the roots while excessive starch accumulation in the stems have been reported in *Ca*. L. asiaticus infected plants previously [4], [6], [8] as well as in this study (Fig. 5, Fig. 7). We could not rule out other possibilities that might contribute to the expression differences between the roots and the stems such as the stems and the roots are in different stages of infection since the plants were graft-transmitted. The collapse and thickening of cell walls in HLB affected roots (Fig. 7 C, D) were not as severe as in the stems (Fig. 5) suggesting that roots were in earlier stage of infection compared to the stems. The early stage of infection of the roots by *Ca*. L. asiaticus probably contributes to that only 111 genes were significantly affected in expression in roots.

The overall expression pattern of the stems to *Ca*. L. asiaticus infection shares high similarity with that of the leaves. High similarity between the expression profiles of the stems and the leaves might be due to that they share some common functions and have some similar phenotypes to *Ca*. L. asiaticus infection. The accumulation of starch, sucrose and glucose as induced by *Ca*. L. asiaticus has been found to be highly concentrated in both the leaves and stems (Fig. 5, Fig. 7) [4], [6], [7]. Collapse of sieve elements of the stems (Fig. 5) was similar to, but not as severe as the leaves (data not shown). The expression pattern of the stems to *Ca*. L. asiaticus infection suggests a very similar virulence mechanism of the pathogen as we learned from the leaf expression pattern including affecting carbohydrate metabolism, phloem blockage and aberrations, cell wall, plant defense, and hormones [58]. The excessive accumulation of starch in the stems (Fig. 7) is due to the similar mechanism as that of the leaves. This accumulation has been attributed to callose deposition, sieve pore plugging by the PP2 protein, and phloem necrosis and collapse [4], [13]–[15], [17], [57]. In addition to the above, other factors, including limited starch breakdown due to the down-regulation of starch-degrading genes, such as amylases and disproportionating enzyme 2 (DPE2), also contribute to the starch accumulation. Starch is degraded by AMY to maltose, and by BMY (BAM) together with DPEs to maltose and glucose. *Ca*. L. asiaticus infection increased the expression of AMY1 but repressed that of BMY1 (Table 1). While AMYs play minor roles, BMYs are indispensable for the breakdown because they catalyze the rate-limiting step [59]. Fan *et al*. [7] linked the accumulation of soluble sugars, such as glucose and fructose, to the up-regulation of cell wall-bound invertases. In our study, expression of the gene encoding Exinv1 was also up-regulated in the stems (Table 1).

Both the expression patterns of the stems and leaves suggest that *Ca*. L. asiaticus infection resemble a susceptible interaction between citrus and the pathogen [4], [13]–[15], [17], [57]. *Ca*. L. asiaticus infection repressed the expression of multiple genes that contain NBS-LRR domains (Table 2). NBS-LRR proteins belong to a major class of disease resistance molecules that recognize pathogenic effectors or plant proteins that are targeted by effectors, and many map to the R (resistance) gene loci [60]. They activate defense signaling [60]. The down-regulated genes encode homologs of the NBS-LRR-like protein cD7, the *Tobacco mosaic virus* (TMV) N-like disease resistance protein [37], and a putative CC-NBS-LRR gene that is related (e-value of 2e-43) to the potato (*Solanum tuberosum*) CC-NB-LRR class gene that was rapidly activated by an incompatible race of *Phytophthora infestans* [61]. The down-regulation of these genes indicates that *Ca*. L. asiaticus infection represses the defense response of citrus, leading to the susceptibility. Interestingly, our previous study indicated that *Ca*. L. asiaticus encodes a functional salicylate hydroxylase (SahA) that converts SA into catechol, which does not induce resistance [58]. SA has been proven to be an endogenous resistance signal, and its derivative MeSA is one of the signals for systemic acquired resistance (SAR). The accumulation of SA and its derivatives are necessary for SAR because it has been shown that plants that are unable to accumulate SA through the transgenic expression of a bacterial salicylate hydroxylase (NahG) that metabolizes SA into catechol are deficient in SAR. The expression of the bacterial salicylate hydroxylase enzyme *in planta* led to decreased SA levels, the failed development of SAR, or the decreased expression of the *PR* genes and heightened susceptibilities to both virulent and avirulent pathogens [62], [63]. On the other hand, *Ca*. L. asiaticus infection also activated the transcription of numerous defense related genes including genes encoding PR10, CC-NB-LRR protein, and disease resistance family protein SC0A (Table 2). However, the induction of plant defense related genes is incapable of preventing the establishment of *Ca*. L. asiaticus in the phloem. Collectively, the expression pattern of plant defense related genes to *Ca*. L. asiaticus infection resemble that of the susceptible plant pathogen interactions [64], [65].

We observed certain expression pattern associated with the stems that was not identified previously with the leaves. For example, *Ca*. L. asiaticus infection repressed expression of genes encoding the pore-forming aquaporins PIP1A, PIP1-1, PIP1-3 while induced expression of SIP1A gene, that belong to the MIP gene family, in the stems (Table 5), where the long distance movement of substances is common. Aquaporins are important molecules in plant physiology because they facilitate the transportation of water and other small, uncharged solutes, such as glycerol, CO2, ammonia and urea, from sources, such as roots and leaves, through the stem to other plant parts [66]–[68]. Their repression in citrus by *Ca*. L. asiaticus infection can deprive affected plants of water and some essential nutrients. Additionally, *Ca*. L. asiaticus infection caused dramatic effect on the phloem (Fig. 5, Fig. 6) in the stems as the major transportation pathway, which might contribute to the disease symptoms in the roots (Fig. 7, Fig. 8). On the other hand, the detrimental effect of HLB on roots will have negative feedback effect on the aerial parts of the plant.

Interestingly, swelling in the middle lamella was observed in both *Ca*. L. asiaticus infected stems and roots, which has not been reported previously (Fig. 6, Fig. 8 ). Swelling in the middle lamella was also observed in the presymptomatic leaf of *Ca*. L. asiaticus infected citrus [8]. The adjacent cells were separate from each other as observed in Fig. 6 and Fig. 8 which might affect nutrient transport among the neighboring cells via plasmodesmata, leading to eventual cell death. However, *Ca*. L. asiaticus does not encode plant cell-wall degradation enzymes such as cellulases, pectinases, xylanases, or endoglucanases [53]. *Ca*. L. asiaticus might indirectly affect the middle lamella by interfering with the plant cell wall related enzymes encoded by citrus such as rhamnogalacturonate lyase which are known to be regulated by plant hormones such as abscisic acid (ABA) and auxins [69]. *Ca*. L. asiaticus was reported to affect plant hormones including IAA and ABA in the fruit [11]. A detailed analysis of hormones in different tissues at different infection stages is required to test this hypothesis in the future. It remains to be determined how *Ca*. L. asiaticus causes the anatomical changes in middle lamella and how those changes affect HLB symptom development.

In conclusion, we analyzed the host response of citrus stems and roots to *Ca*. L. asiaticus infection. Dramatic differences in the transcriptional responses were observed between the citrus stems and roots to *Ca*. L. asiaticus infection. Microscopy analyses indicated that *Ca*. L. asiaticus infection significantly affects the starch accumulation, and sieve elements of the stems or roots. Understanding how to reduce the adverse effect of *Ca*. L. asiaticus infection on the leaves, stems and roots is critical to manage HLB.

## Materials and Methods

### Source of Plant Materials

Two-year-old Valencia sweet orange (*C. sinensis*) on rootstock Swingle citrumelo (*Citrus paradisi*. Macf. × *Poncirus trifoliata* [L.] Raf.) plants used in this study were graft-inoculated with budwood from *Ca*. L. asiaticus infected citrus trees and maintained in a USDA-APHIS/CDC-approved secured greenhouse. The inoculated plants that were used in this experiment were *Ca*. L. asiaticus-free before the graft inoculations, as shown by PCR and Q-PCR tests using specific primers [12]. Stem and root samples were obtained from three HLB symptomatic trees and three healthy control trees of similar size and from similar positions approximately 16 months after inoculation. The presence of *Ca*. L. asiaticus in the plants was confirmed using both conventional and quantitative PCR as described previously [12].

### Microarray Analysis

Total RNA from the stems and roots were isolated from freshly obtained samples using the RNeasy Plant Mini Kit and treated with DNase (Qiagen, Valencia, CA). Root samples were prepared by excising small pieces from lateral roots and were frozen in liquid nitrogen before RNA purification. Stem pieces were harvested from young stems bearing symptomatic leaves by peeling off the bark and phloem together. For the softer stem parts, the whole stem was cut into smaller pieces and processed, as described above for the roots. The samples were grinded in liquid nitrogen with a mortar and pestle, the powder was rapidly suspended in RLT buffer (Qiagen, Valencia, CA) that was supplemented with 1% mercaptoethanol, and the solution was processed using the RNeasy Plant Mini Kit according to the manufacturer’s instructions. The quality of RNA was checked using the NanoDrop™ 1000 spectrophotometer (NanoDrop Technologies, Inc.), and only samples with A260/280 and A260/230 nm ratios of ∼2.0 were selected. The integrity of the total RNA was further determined using the Agilent 2100 Bioanalyzer (Agilent Technologies, Santa Clara, CA).

Microarray experiments were carried out at the Gene Expression Core Facility of the Interdisciplinary Center for Biotechnology Research at the University of Florida. Data analyses were conducted as described previously [4]. Briefly, the Affymetrix GeneChip was used for microarray analysis. The GeneChip Citrus Genome Array contains 30,171 probe sets representing up to 33,879 citrus transcripts based on EST sequences obtained from several citrus species and citrus hybrids. The raw data were normalized using the robust multichip analysis (RMA) approach [70]. Linear models were used to assess the differential expression, while the empirical Bayes method was used to moderate the standard errors [71]. Differentially expressed genes were ranked by P values and fold changes. Genes with a cutoff threshold P value of 0.05 and LFC of ≥1.00 or ≤−1.00 were considered to be differentially expressed. To generate diagrams of the metabolic pathways and biological processes that were regulated, we used the Open Source MapMan 3.5.0 BETA program [24]. The gene ontology system in the MapMan program was used for the identification of the processes, pathways and gene families whose expression were significantly altered. Details of our microarray experiments and the MIAME-compliant microarray data have been deposited in the Gene Expression Omnibus database, the National Center of Biotechnology Information (Accession Number GSE33004).

### Quantitative Reverse Transcription PCR (qRT-PCR) Assays

Expression of seven genes was confirmed using qRT-PCR and the same set of RNA that was used for the microarray. Primers were designed using the PrimerQuest^SM^ program (Integrated DNA Technologies, Inc). All of the qRT-PCR reactions were performed using total volumes of 25 µl in an ABI PRISM 7900 sequence detection system (Applied Biosystems, Foster City, CA) with the QuantiTect SYBR Green RT-PCR Kit (Qiagen, Valencia, CA) and ∼50 ng of total RNA, 10 nM PCR primers, 0.25 µl RT mix (Qiagen, Valencia, CA) and 12.5 µl QuantiTect SYBR Green RT-PCR Master Mix (Qiagen, Valencia, CA). The PCR conditions were 30 min of reverse transcription at 50°C followed by 15 min of predenaturation at 95°C and 40 cycles of 15 s of denaturation at 94°C, 45 s of annealing at 55°C, and 30 s of extension at 72°C. The 18S rRNA gene was used as an endogenous control. At the end of the cycling phase, a dissociation curve was produced to ensure the specificity of the amplification. The relative expression ratios for each target gene were calculated using the 2^–ΔΔCt^ method according to Livak and Schmittgen [72]. Details of the primers that were used and the genes that were tested are listed in Table 7.

### Microscopy Analysis

Healthy and HLB affected round to triangular shaped young stems and roots showing primary and secondary growth were prepared for light and transmission electron microscopy using the following method. Samples were placed in 3% glutaraldehyde in 0.1 M potassium phosphate buffer, pH 7.2 for 4 hr at room temperature followed by 3 washes in the same buffer. Post-fixation in 2% osmium tetroxide in the same buffer was carried out for 4 hr at room temperature, then washed in two buffer changes before dehydration in acetone at 10% for 10 min each step. The samples were then infiltrated and embedded in Spurr’s resin [73]. One micrometer cross sections were made on an LKB Huxley Ultramicrotome (LKB Instruments Inc., Rockville, MD, USA) and stained with methylene blue/azure A, counter-stained with basic fuchsin [74]. These were photographed using a Leitz Labor-Lux S compound microscope (Leitz, Wetzlar, Germany) equipped with a Canon Power Shot S31 S digital camera. The same blocks were then thin sectioned with the same microtome, mounted on 200 mesh Formvar coated Cu grids followed by staining with 2% aq. uranyl acetate and post staining with Reynolds lead citrate [75]. The grids were examined with a Morgagni 268 transmission electron microscope (FEI, The Netherlands) equipped with an AMT digital camera (Danvers, MA, USA).

## Supporting Information

Figure S1 **Cellular pathways that are regulated by** ***Ca*** **. L. asiaticus infection in the stems and roots of Valencia sweet orange (** ***Citrus sinensis*** **).** A = stem and B = root. Genes that were significantly up-regulated following *Ca*. L. asiaticus infection are displayed in blue, and down-regulated genes are displayed in red.
(TIF)

Figure S2 **Regulatory pathways that are altered by** ***Ca*** **. L. asiaticus infection in the stems and roots of Valencia sweet orange (** ***Citrus sinensis*** **).** A = stem and B = root. Genes that were significantly up-regulated following *Ca*. L. asiaticus infection are displayed in blue, and down-regulated genes are displayed in red.
(TIF)

Figure S3 **Starch and sugar metabolic pathway genes that are regulated by** ***Ca*** **. L. asiaticus infection in the stems of Valencia sweet orange (** ***Citrus sinensis*** **).** Genes that were significantly up-regulated following *Ca*. L. asiaticus infection are displayed in blue, and down-regulated genes are displayed in red. Abbreviations/definitions: ADP-glucose pyrophosphorylase large subunit 3 (APL3), granule-bound starch synthase (GBSS), acid invertase (ACI), vacuolar invertase (VAI), alpha-amylase (AMY), beta-amylase (BMY), and sugar transporter 1 (SUT1).
(TIF)

Figure S4 **Regulation of transcription factor-encoding genes by** ***Ca*** **. L. asiaticus infection in the stems and roots of Valencia sweet orange (** ***Citrus sinensis*** **).** A = stem and B = root. Genes that were significantly up-regulated following *Ca*. L. asiaticus infection are displayed in blue, and down-regulated genes are displayed in red. Abbreviations/definitions: ABI3/VP1, ABI3/VP1-related B3-domain-containing TF family; AP2/EREBP, APETALA2/ethylene-responsive element binding protein family; ARF, auxin response factor; bZIP, basic leucine zipper motif; bHLH, basic helix-loop-helix family; C2C2-CO-like, CONSTANS-like zinc finger family; C2C2-Dof, C2C2(Zn) Dof family; C2C2-YABBY, C2C2(Zn) YABBY family; C2C2-GATA, C2C2(Zn) GATA family; C2H2, C2H2 zinc finger family; C3H, C3H zinc finger family; CCAAT-DR1, CCAAT box binding factor DR1; ORPHAN, Orphan family; NAC, NAC domain; MYB-related, MYB-related family; MYB, MYB domain; MADS, MADS box domain; HB, homeobox TF family; HSF, heat shock TF family; GRF, GRF family; G2-like, G2-like family GARP; GRAS, GRAS family; E2F-DP, E2F/DP family; EIL, EIN3-like; CCAAT-HAP2, CCAAT box binding factor HAP2; CCP, CPP(Zn), CPP1-related family; SBP, SBP family; TCP, TCP domain TF; Global, Global TF group; High mobility, high mobility group (HMG) family; Trihelix, triple helix family; TUB, Tubby (TUB) homolog TF; Histone DAase, histone deacetylase; Histone ATse, histone acetyltransferase; WRKY, WRKY domain family; AS2, lateral organ boundary gene family; JUMONJI, JUMONJI class TF; AT-rich, AT-rich interaction domain-containing family; AtSR, AtSR family; LUG, LEUNIG (LUG) domain family; Methyl BD, methyl binding domain proteins; Aux/, Aux/family; B3, B3 DNA binding domain TF; NPR1, NPR1 family; NIN-like, NIN-like bZIP-related family; Bromodomain, bromodomain proteins; BZR, Brassinazole resistant TF family; Nucleosome assembly, nucleosome/chromatin assembly factor group; Chromatin Remodeling, chromatin remodeling factors; PHD finger, PHD finger family; PHOR1, photoperiod-responsive 1; Dicer-like, dicer; DNA MT, DNA methyltransferase; Polycomb, Polycomb group (PcG); Pseudo ARR, Pseudo ARR; ELF, ELF3 TF; FHA, Forkhead-associated (FHA) domain TF family; PWWP domain, PWWP domain protein family; SET-domain, SET-domain transcriptional regulator family; GeBP, GeBP-like family; General, general transcription; Silencing, silencing group; SNF7, SNF7 family; TAZ, Transcriptional co-activator with PDZ binding motif; CCHC, Zn-finger (CCHC).
(TIF)

Figure S5 **Regulation of secondary metabolic pathway genes by** ***Ca*** **. L. asiaticus infection in the stems and roots of Valencia sweet orange (** ***Citrus sinensis*** **).** A = stem and B = root. Genes that were significantly up-regulated following *Ca*. L. asiaticus infection are displayed in blue, and down-regulated genes are displayed in red.
(TIF)

Figure S6 **Regulation of phenylpropanoid pathway genes by** ***Ca*** **. L. asiaticus infection in the stems of Valencia sweet orange (** ***Citrus sinensis*** **).** Genes that were significantly up-regulated following *Ca*. L. asiaticus infection are displayed in blue, and down-regulated genes are displayed in red. There was no significantly up-regulated phenylpropanoid pathway genes observed in the roots.
(TIF)
