# Supplementary figures and images for: Transcriptional and Microscopic Analyses of Citrus Stem and Root Responses to Candidatus Liberibacter asiaticus Infection
Source: PLoS One. 2013 Sep 13;8(9):e73742. doi: 10.1371/journal.pone.0073742 (PMC3772824; doi:10.1371/journal.pone.0073742)

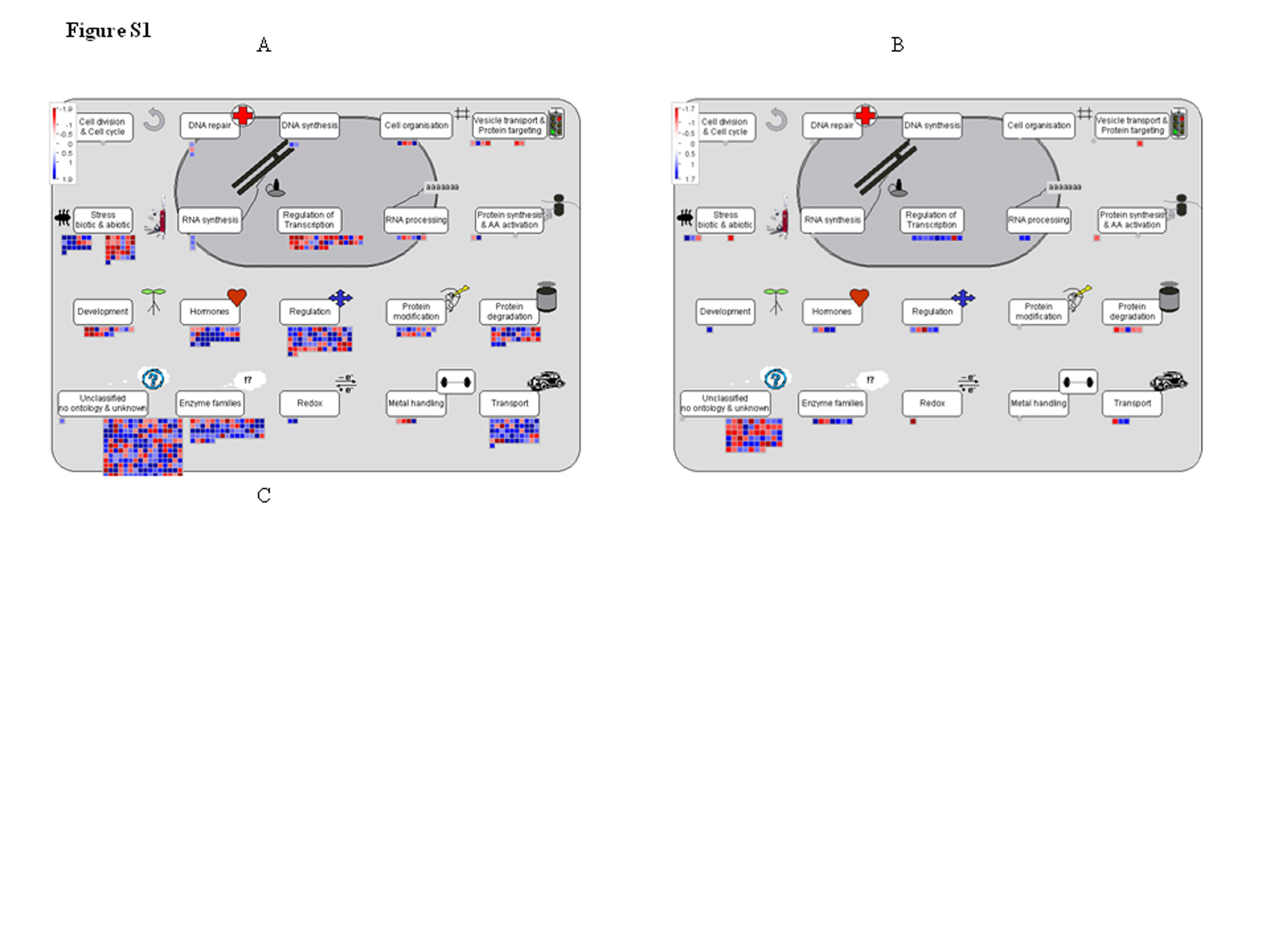

Supplement: Figure S1 — Cellular pathways that are regulated by Ca . L. asiaticus infection in the stems and roots of Valencia sweet orange ( Citrus sinensis ). A = stem and B = root. Genes that were significantly up-regulated following Ca. L. asiaticus infection are displayed in blue, and down-regulated genes are displayed in red. (TIF) [file pone.0073742.s001.tif]

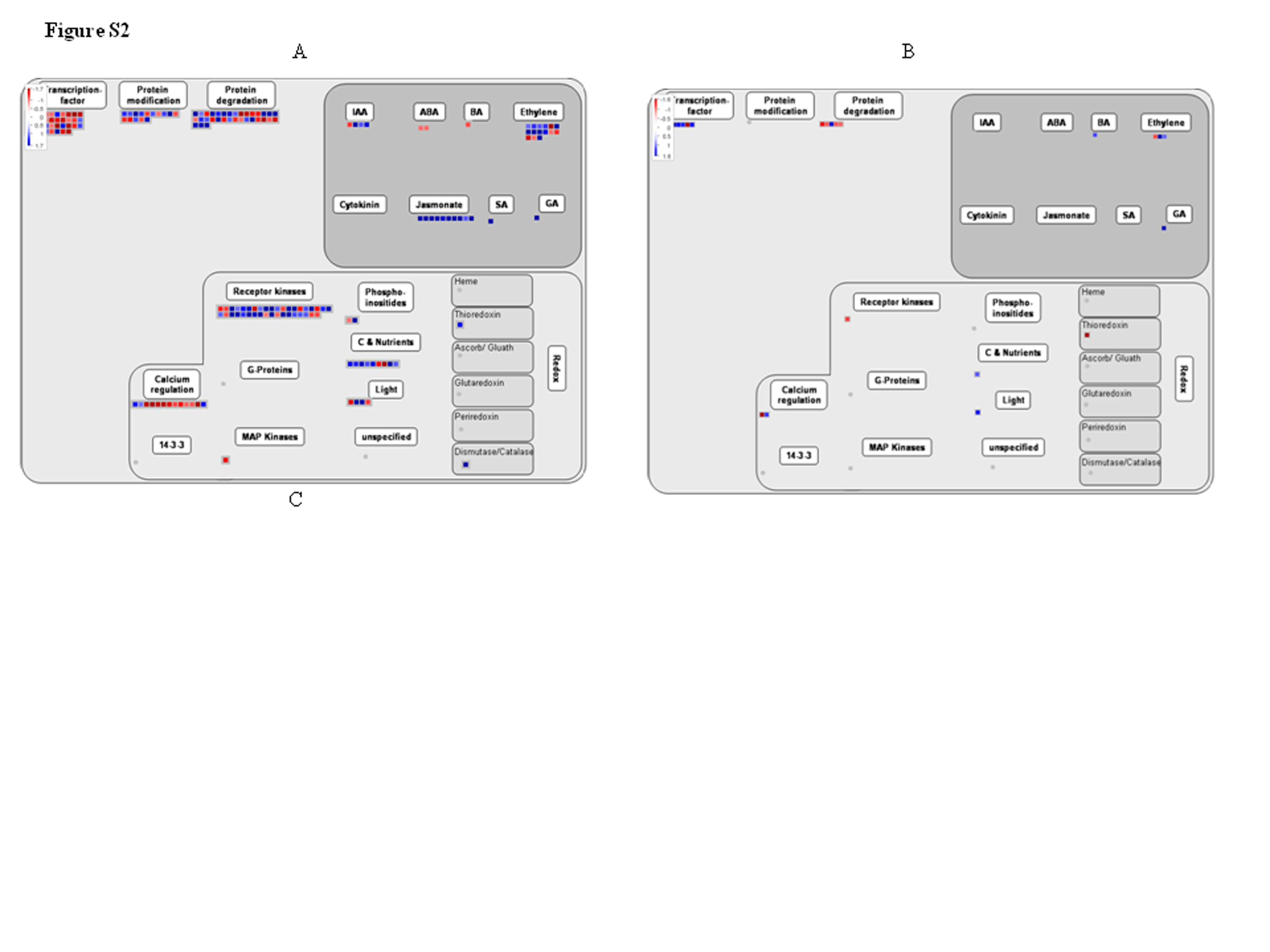

Supplement: Figure S2 — Regulatory pathways that are altered by Ca . L. asiaticus infection in the stems and roots of Valencia sweet orange ( Citrus sinensis ). A = stem and B = root. Genes that were significantly up-regulated following Ca. L. asiaticus infection are displayed in blue, and down-regulated genes are displayed in red. (TIF) [file pone.0073742.s002.tif]

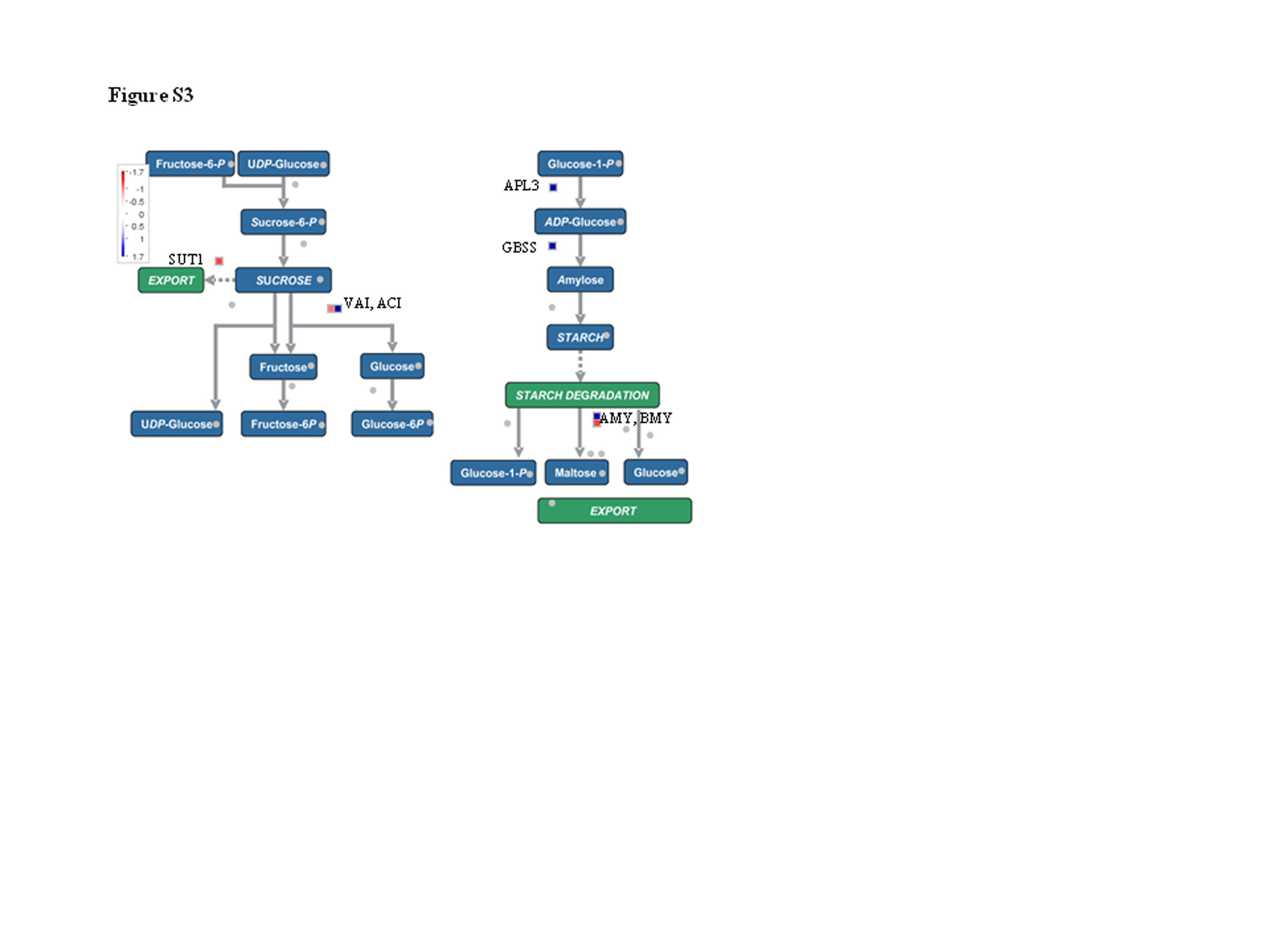

Supplement: Figure S3 — Starch and sugar metabolic pathway genes that are regulated by Ca . L. asiaticus infection in the stems of Valencia sweet orange ( Citrus sinensis ). Genes that were significantly up-regulated following Ca. L. asiaticus infection are displayed in blue, and down-regulated genes are displayed in red. Abbreviations/definitions: ADP-glucose pyrophosphorylase large subunit 3 (APL3), granule-bound starch synthase (GBSS), acid invertase (ACI), vacuolar invertase (VAI), alpha-amylase (AMY), beta-amylase (BMY), and sugar transporter 1 (SUT1). (TIF) [file pone.0073742.s003.tif]

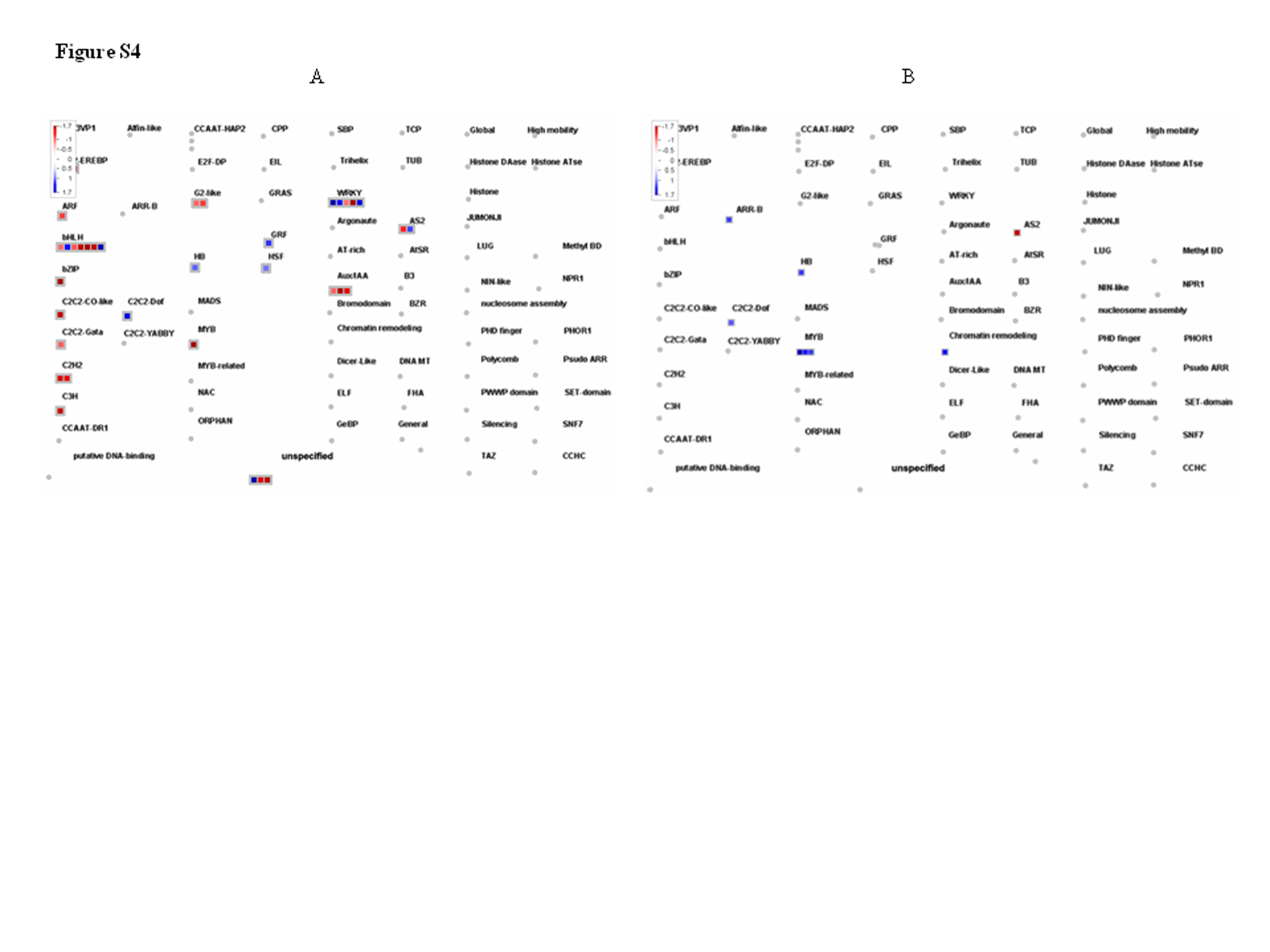

Supplement: Figure S4 — Regulation of transcription factor-encoding genes by Ca . L. asiaticus infection in the stems and roots of Valencia sweet orange ( Citrus sinensis ). A = stem and B = root. Genes that were significantly up-regulated following Ca. L. asiaticus infection are displayed in blue, and down-regulated genes are displayed in red. Abbreviations/definitions: ABI3/VP1, ABI3/VP1-related B3-domain-containing TF family; AP2/EREBP, APETALA2/ethylene-responsive element binding protein family; ARF, auxin response factor; bZIP, basic leucine zipper motif; bHLH, basic helix-loop-helix family; C2C2-CO-like, CONSTANS-like zinc finger family; C2C2-Dof, C2C2(Zn) Dof family; C2C2-YABBY, C2C2(Zn) YABBY family; C2C2-GATA, C2C2(Zn) GATA family; C2H2, C2H2 zinc finger family; C3H, C3H zinc finger family; CCAAT-DR1, CCAAT box binding factor DR1; ORPHAN, Orphan family; NAC, NAC domain; MYB-related, MYB-related family; MYB, MYB domain; MADS, MADS box domain; HB, homeobox TF family; HSF, heat shock TF family; GRF, GRF family; G2-like, G2-like family GARP; GRAS, GRAS family; E2F-DP, E2F/DP family; EIL, EIN3-like; CCAAT-HAP2, CCAAT box binding factor HAP2; CCP, CPP(Zn), CPP1-related family; SBP, SBP family; TCP, TCP domain TF; Global, Global TF group; High mobility, high mobility group (HMG) family; Trihelix, triple helix family; TUB, Tubby (TUB) homolog TF; Histone DAase, histone deacetylase; Histone ATse, histone acetyltransferase; WRKY, WRKY domain family; AS2, lateral organ boundary gene family; JUMONJI, JUMONJI class TF; AT-rich, AT-rich interaction domain-containing family; AtSR, AtSR family; LUG, LEUNIG (LUG) domain family; Methyl BD, methyl binding domain proteins; Aux/, Aux/family; B3, B3 DNA binding domain TF; NPR1, NPR1 family; NIN-like, NIN-like bZIP-related family; Bromodomain, bromodomain proteins; BZR, Brassinazole resistant TF family; Nucleosome assembly, nucleosome/chromatin assembly factor group; Chromatin Remodeling, chromatin remodeling factors; PHD finger, PHD finger family; PHOR [file pone.0073742.s004.tif]

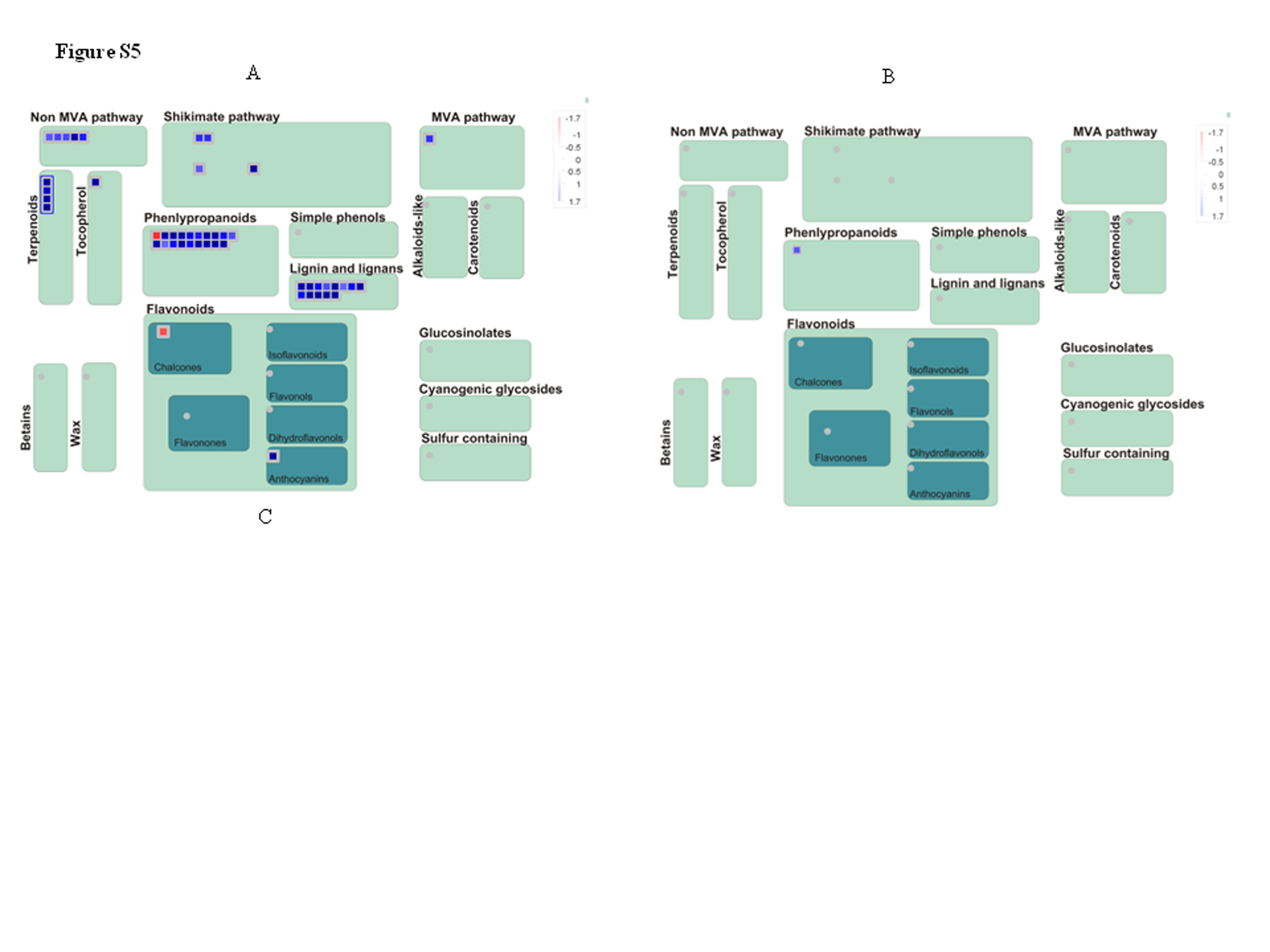

Supplement: Figure S5 — Regulation of secondary metabolic pathway genes by Ca . L. asiaticus infection in the stems and roots of Valencia sweet orange ( Citrus sinensis ). A = stem and B = root. Genes that were significantly up-regulated following Ca. L. asiaticus infection are displayed in blue, and down-regulated genes are displayed in red. (TIF) [file pone.0073742.s005.tif]

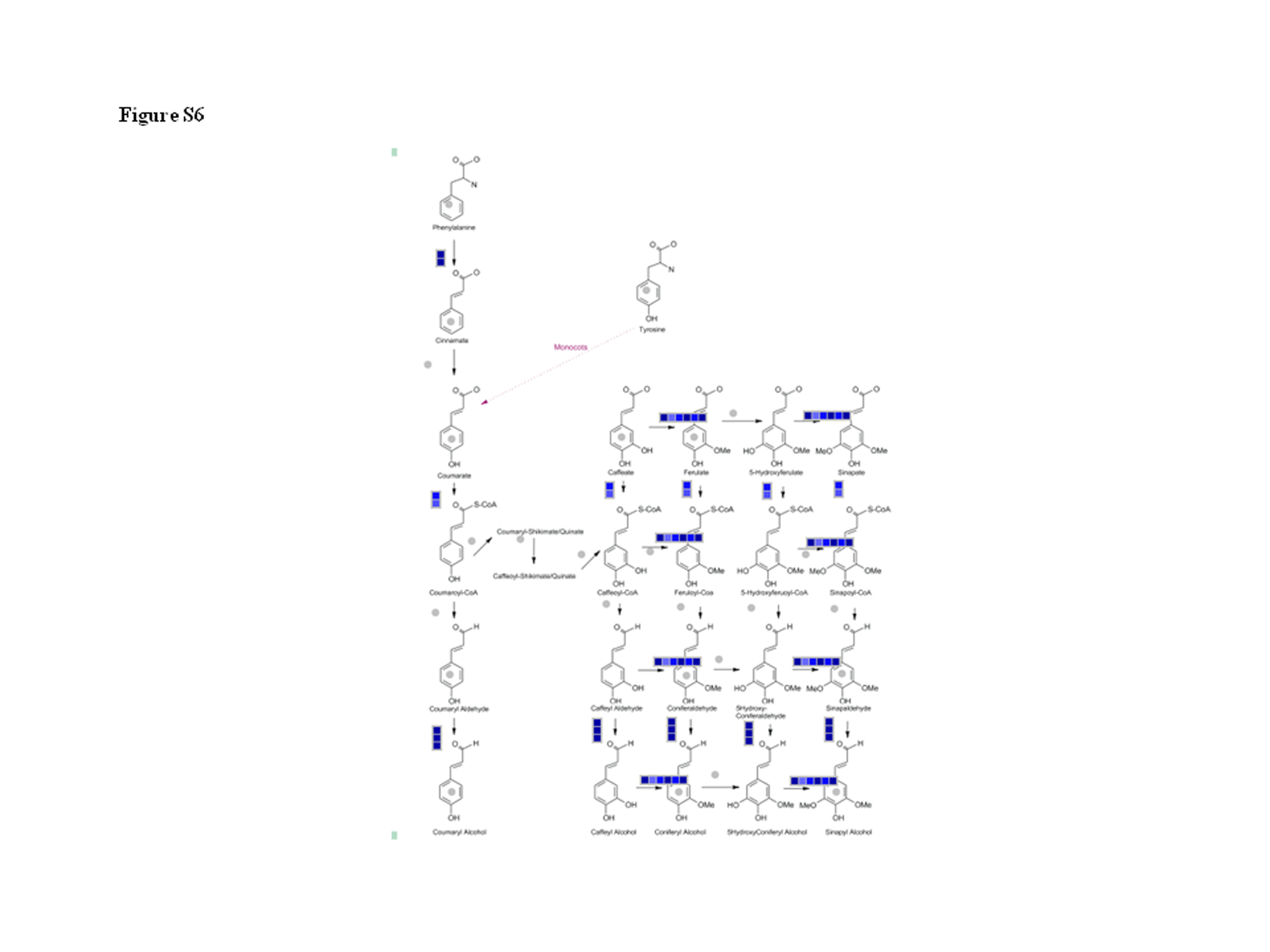

Supplement: Figure S6 — Regulation of phenylpropanoid pathway genes by Ca . L. asiaticus infection in the stems of Valencia sweet orange ( Citrus sinensis ). Genes that were significantly up-regulated following Ca. L. asiaticus infection are displayed in blue, and down-regulated genes are displayed in red. There was no significantly up-regulated phenylpropanoid pathway genes observed in the roots. (TIF) [file pone.0073742.s006.tif]
